# METTL1 promotes cadmium-induced stress granules formation via enhancing translation of G3BP1 and expression of m^7^G- 3' tiRNA Met^CAT^

**DOI:** 10.1007/s10565-025-10072-0

**Published:** 2025-08-05

**Authors:** Wenyu Hu, Yaomin Liang, Xiaoling Ying, Yapeng Huang, Chang Xiong, Bixia Liu, Yifan Lv, Cong Chen, Chengcheng Zhang, Haiqing Zhang, Hu Li, Mei Yang, Weidong Ji

**Affiliations:** 1 Center for Translational Medicine, The First Affiliated Hospital, Sun Yat-Sen University , Guangzhou, 510080 China; 2 Department of Urology, The Second Affiliated Hospital of Guangzhou Medical University, Guangzhou, 510220 China; 3 Department of Cardiology, Guangdong Provincial People’s Hospital, Guangzhou, 510080 China; 4 Department of Urology, The First Affiliated Hospital of Guangzhou Medical University, Guangzhou, 510230 China; 5 Department of Gynecology, The First Affiliated Hospital, Ji Nan University, Guangzhou, 519070 China; 6 Department of Breast Surgery, Zhujiang Hospital, Southern Medical University, Guangzhou, 510080 China

**Keywords:** METTL1, Stress granules, G3BP1, TsRNAs, Cadmium

## Abstract

Methyltransferase 1 (METTL1) is currently regarded as a key tRNA m^7^G writer. Recent studies indicate its potential role in carcinogenesis via increased m^7^G modification to stabilize tRNA and upregulate tRNA expression. Cadmium-induced stress triggers the assembly of stress granules (SGs) and production of tRNA-derived stress-induced RNAs (tiRNAs). However, whether METTL1 is involved in the formation of cadmium-induced SGs and its mechanism are still unclear. Here, we demonstrated that METTL1 promotes cadmium-induced SGs formation. Mechanistically, METTL1 not only upregulates SG's core protein Ras-GTPase-activating protein SH3 domain-binding protein 1 (G3BP1) translation through tRNAs m^7^G modification, but also enhances expression of one m^7^G-modified tiRNA, m^7^G-3' tiRNA-Met^CAT^ (mtiRM), which affects SGs assembly. Together, the findings concluded that the promotional effect of METTL1 on cadmium-induced SGs formation jointly through G3BP1 translation and mtiRM expression, thus providing insights into an intimate link between SGs and tumorigenesis.

**Supplementary Information:**

The online version contains supplementary material available at 10.1007/s10565-025-10072-0.

## Introduction

In recent years, the role of RNA epigenetic regulation in the occurrence and development of tumors has attracted increasing attention. The m^7^G modification is widely present in various organisms and is one of the most conserved modifications (Edmonds et al. 1991). The m^7^G modification is mediated by the METTL1/WDR4 complex, which consists of the methyltransferase 1 (METTL1) and its auxiliary protein WD Repeat Domain 4 (WDR4) (Alexandrov et al. 2002), and is found in mRNAs (Ramanathan et al. 2016), tRNAs (Lin et al. 2018), and rRNAs (White et al. 2008). For mammalian tRNAs, the m^7^G modification is commonly mediated by METTL1/WDR4 complex at the 46 positions of the variable loop, stabilizing tRNA’s tertiary structure and preventing tRNA degradation (Jovine et al. 2000; Orellana et al. 2021). Studies have shown that the absence of METTL1 leads to reduced tRNA methylation levels, abnormal translation, and cell cycle defects, while overexpression of METTL1/WDR4 leads to malignant transformation and tumorigenesis (Guzzi and Bellodi 2020; Xia et al. 2023).

tRNA fragmentation upon various stress stimuli have also been observed (Kumar et al. 2016). These 18–40 nucleotide (nt) sncRNAs can be roughly divided into two categories based on their sequence and biogenesis: tRNA-derived fragments (tRFs) and tRNA-derived stress-induced RNAs (tiRNAs) (Liang et al. 2024; Ma et al. 2020). The majority of tiRNAs are produced by stress-induced angiogenin (ANG), which mediates specific cleavage of the anticodon loop of tRNA, including 5'tiRNAs and 3'tiRNAs (Emara et al. 2010). Concrete evidence suggests that tRNA modifications not only have an impact on tRNA function and abundance, but dysregulation of tRNA modification alters the tsRNA abundance in human cells (Liang et al. 2024). Of note, tsRNA carries a variety of RNA modifications inherited from their overwhelmingly modified tRNA precursors (Liang et al. 2024). Our previous study identified a novel m^7^G-modified tsRNA, which is highly expressed in cadmium chloride (CdCl_2_)-induced SV-HUC-1 malignant transformed cells and in a CdCl_2_-induced rat bladder cancer model. It promotes the occurrence and progression of bladder cancer (Ying et al. 2024). However, the mechanism of m^7^G-modified tsRNAs in the stress effect during the early stages of cadmium has not yet been elucidated.

It’s known that the activation of stress response pathways drives both tsRNA production or stress granule (SGs) formation. SGs are membrane-less organelles that form in the cytoplasm during cellular stress responses involving in stress adaptation mechanism (Namkoong et al. 2018). SGs regulate mRNA storage, stability, and translation (Ivanov et al. 2019). They also be associated with various diseases such as neurodegenerative disease, tumorigenesis and drug resistance (Gao et al. 2019; Li et al. 2013; Zhao et al. 2023). Internal SGs contain abundant RNAs and RNA-interacting protein networks, among the crucial components which a ‘core SGs network’ comprising 36 proteins that strongly influences SGs assembly, with G3BP1 and G3BP2 proteins at the core of this network, acting as switches for SGs assembly under arsenite stress (Yang et al. 2020). In stress, a large number of mRNAs with internal m^7^G modifications, mediated by METTL1 are recruited to SGs, and METTL1 knockdown significantly reduces the abundance of m^7^G modifications within SGs, which affecting mRNA stability and translation efficiency (Zhao et al. 2023).

In this study, we investigated the effect and mechanism of METTL1-mediated m^7^G modification on cellular stress in acute exposure to cadmium. We revealed that METTL1 promotes the formation of SGs induced by CdCl_2_ in bladder urothelial cells and BC cells. We discover METTL1 enhance the translation of the SGs core protein G3BP1, which is conducive to the formation of SGs. Secondly, we identified a novel m^7^G-modified tsRNA (3' tiRNA Met^CAT^) that is mediated by METTL1. METTL1 facilitates the formation of SGs by mediating the m^7^G modification of 3' tiRNA Met^CAT^. This finding not only provides an important theoretical basis for revealing the RNA epigenetic mechanisms of SGs, but also offers new perspectives for the study of RNA epigenetic prevention and treatment of cadmium.

## Materials and methods

### Cell lines and cell culture

Human immortalized uroepithelial cells SV-HUC-1, 293T cells and human bladder cancer cells T24 were purchased from the American Type Culture Collection (Manassas, VA, USA) and the Shanghai Institute of Cell Biology, Chinese Academy of Sciences (Shanghai, China). In this study, cells were cultured using complete media containing 10% fetal bovine serum (FBS) and 100 U/ml penicillin–streptomycin. SV-HUC-1 cells were cultured in Ham's F-12 K (Kaighn's) medium (Gibco, USA), T24 cells were cultured in RPMI-1640 medium (Gibco, USA), and 293T cells were cultured in DMEM (Gibco, USA) medium. The cell culture parameters were set to 5% CO_2_ and 37 °C. All cell lines were free of mycoplasma infection.

### Stressors and treatment

CdCl_2_ (Sigma, USA) and NaAsO_2_ (Sigma, USA) were dissolved in ddH_2_O to a concentration of 10 μmol/ml and stored long-term at −80 °C. Before treating cells, fresh culture medium was used to dilute CdCl_2_ and NaAsO_2_ to the required concentrations, pre-warmed to 37 °C, and then added to the cells for culture for an appropriate amount of time.

### Immunofluorescence and statistical analysis of SGs

Cells were grown in 8-well chamber slides (Millipore, USA). Add 300 M CdCl_2_/NaAsO_2_ for 1 h stimulation. Subsequently, the cells were washed three times with PBST buffer (0.1% Tween) at 37 ℃ for 5 min each time. The cells were then fixed with 4% paraformaldehyde (Sihe Biotech, China) at room temperature for 20 min, followed by three washes with PBST buffer. The cells were permeabilized with 0.5% Triton X-100 for 5 min, and then washed three times with PBST buffer. Next, blocked with 1% bovine serum albumin (BSA) for 1 h. Cells were further incubated with primary antibodies in 1% BSA overnight at 4 ℃, followed by three washes with PBST buffer. Then, cells were incubated with secondary antibody at room temperature for 1 h, avoiding exposure to light. After three washes with PBST buffer, the cells were incubated with DAPI for 10 min and then washed three times with PBST buffer, avoiding exposure to light. Images were captured by ZEISS/LSM880 confocal microscope (Zeiss, Germany).

To perform statistical analysis on the photographed images, the positive cell rate for the production of SGs can be calculated using the following formula: $Positive\;Cell\;Rate=\frac{Number\;of\;cell\;producing\;SGs}{Total\;number\;of\;cells}\times100\%$. The antibodies used are shown in Table S1(Supplementary file 1).

### Plasmid construction and mutagenesis assay

The cell lines for overexpression/knockdown of METTL1 have been previously constructed in the laboratory (Ying et al. 2021). The plasmid for overexpressing G3BP1-EGFP was obtained by cloning the target gene G3BP1-EGFP cDNA from the phage UbiC G3BP1-EGFP-EGFP plasmid (Addgene 119950) and then ligating it with LentiORF PLEX-MCS. The primers used are listed in Table S2(Supplementary file 1). The G3BP1 mutant fragment was synthesized by Azenta Life Sciences, and the synthesis report is provided in Supplementary File 7. The cDNA of G3BP1 wild-type and mutant were ligated into 2AB-Flag vector. For detailed experimental methods, please refer to Supplementary File 1.

### Western blot

The cells were lysed with RIPA buffer (Beyotime, China) containing 1 × PMSF. After determining the protein concentration using the BCA protein assay kit (Beyotime, China), the protein sample was mixed with 5 × SDS-PAGE loading buffer (Beyotime, China) and boiled at 100℃ for 10 min. The sample was separated by 10% SDS-PAGE gels and transferred onto a polyvinylidene difluoride membranes (Biorad, USA). The membrane was blocked with 5% BSA at room temperature for 1 h, and then incubated with the primary antibody overnight at 4 ℃. After washing the membrane three times with TBST (0.1% Tween) for 10 min each time, the membrane was incubated with the secondary antibody at room temperature for 1 h and then washed three times with TBST. The immune response was visualized by using ECL chemiluminescence reagent kit (Thermo Fisher, USA), and the image was captured using iBright™ FL1500 Imaging System (Thermo Fisher, USA). The antibodies used are shown in Table S1.

### RNA isolation and qRT-PCR

After extracting total cellular RNA using Trizol reagent (Invitrogen, USA), the RNA was reverse transcribed into cDNA using PrimeScript™ RT Reagent Kit with gDNA Eraser (TaKaRa, Japan). According to the manufacturer’s instructions, a mixture containing mix, cDNA, and primers were prepared. The cDNA in the mixture was quantified using a Step-One Fast Real-time PCR System(Applied Biosystems, USA), and the data were calculated using the 2 ^-△△CT^ method. The primers used are listed in Table S2.

### Sucrose gradient centrifugation and polysome fractionation

6 × 10^7^ cells were added 100 μg/mL CHX (Sigma, USA) and cultured at 37 °C for 15–20 min. Then the cells were washed twice with pre-cooled PBS and collected in a centrifuge tube. The tube was put on ice and cells were lysed with polysome cell extraction buffer (50 mM MOPS, 150 mM NaCl, 15 mM MgCl_2_, 0.5% Triton X-100, 100 μg/ml Cycloheximide, 200 U RNaseOUT, 1 mg/ml Heparin, 2 mM PMSF, and 1 μM Benzamine) for 10 min. After centrifugation (12,000 × rpm, 15 min, 4 °C), supernatant was collected and added to a 10%−50% sucrose gradient prepared with sucrose and polysome buffer (0.05 M MOPS, 0.015 M MgCl_2_, 0.15 M NaCl, 0.1 mg/mL CHX). Monosomes and polysomes were separated by using the SW41 rotor (Beckman Coulter, USA) to centrifuge at 36,000 rpm for 2.5 h at 4 °C. mRNA was purified and reversed transcription, then an equal volume of cDNA for qRT-PCR analysis.

### Extraction for SG core RNA

Extraction for SG core RNA was performed as previously described (Khong et al. 2018). In brief, 6 × 10^7^ cells were cultured in fresh pre-warmed culture medium containing 300 μM CdCl_2_ at 37 °C for 1 h. After that, the cells were washed three times with fresh pre-warmed culture medium at 37 °C and collected in a 50 mL Eppendorf tube. The tube was centrifuged at 1500 g for 3 min and the supernatant was discarded. The cell pellet was then transferred to liquid nitrogen for rapid freezing overnight. The next day, the cell pellet was thawed on ice for 30 min and SG lysis buffer was added to fully lyse the cell pellet. The supernatant (SG core-enriched fraction) was collected through multiple centrifugations. The SG-enriched fraction was pre-treated with Pierce™ Protein A/G Magnetic Beads (Thermo Fisher, USA) balanced with SG lysis buffer containing DEPC. Subsequently, GFP antibody was added and incubated overnight at 4 °C with rotation. The mixture was then centrifuged at 18,000 × g for 20 min at 4 °C, the supernatant was removed, and magnetic beads was added. The mixture was rotated at 4 °C for 180 min. The beads were washed with Wash buffer 1, Wash buffer 2 and Wash buffer 3, followed by resuspension in 250 μL of 1 × Proteinase K buffer and incubated at 37 °C for 15 min. Finally, 750 μL of Trizol was added to purify the SG core RNA. The components of the buffers used are provided in Supplementary Material 1.

### tRF & tiRNA sequencing

The tRF & tiRNA sequencing was conducted by Kangcheng Biotech (Guangzhou, China). Purified RNA was extracted and assessed for quality using the NanoDrop ND-1000 spectrophotometer (Thermo Fisher, USA). To eliminate RNA modifications that could interfere with small RNA-seq library construction, total RNA samples were subjected to the following pretreatments: deacylation of 3'-aminoacyl (charged) groups to generate 3'-OH for 3'-adapter ligation; removal of 3'-cP (2',3'-cyclic phosphate) to produce 3'-OH for 3'-adapter ligation; phosphorylation of 5'-OH (hydroxyl group) to create 5'-P for 5'-adapter ligation; and demethylation of m^1^A and m^3^C to facilitate efficient reverse transcription. Library preparation involved ligating 3' and 5' small RNA adapters to both ends of the total RNA from each sample, followed by synthesis and amplification of cDNA using Illumina-specific RT reverse transcription primers and amplification primers. Subsequently, PCR amplicons of ~ 134-160 bp were extracted and purified from PAGE gel. Finally, the completed libraries were quantified using the Agilent 2100 Bioanalyzer. The libraries were denatured and diluted to a loading concentration of 1.8 pM in a final volume of 1.3 mL. The diluted libraries were then loaded onto the reagent cartridge according to the Illumina NextSeq 500/550 V2 Kit (FC-404–2005, Illumina, USA) and run on the Illumina NextSeq 500 system (Illumina, USA). Data were collected and analyzed.

Image analysis and base calling are performed using Solexa pipeline v1.8 (Off-Line Base Caller software, v1.8). Sequencing quality are examined by FastQC (Andrews 2010) and trimmed reads (pass Illumina quality filter, trimmed 5, 3 -adaptor bases by cutadapt (Martin 2011)) are aligned allowing for 1 mismatch only to the mature tRNA sequences, then reads that do not map are aligned allowing for 1 mismatch only to precursor tRNA sequences with bowtie software (Langmead et al. 2009). The remaining reads are aligned allowing for 1 mismatch only to miRNA reference sequences with miRDeep2 (Friedländer et al. 2012). The expression profiling of tRF & tiRNA and miRNA can be calculated based on counts of reads mapped. The differentially expressed tRFs & tiRNAs are screened based on the count value with R package edgeR (Robinson et al. 2010).

### tiRNAs were detected by qRT-PCR

Total RNA from cells was extracted using Trizol reagent. According to the rtStar™ tRF & tiRNA Preatreatment Kit (Arraystar, USA) manufacturer’s instructions, the total RNA was pre-treated, including 3' terminal deacetylation, demethylation, 3'-cP removal, 5'-P addition, and 5' adaptor ligation. Following the PrimeScript™ RT reagent Kit (Perfect Real Time) (Takara, Japan) manufacturer’s instructions, the pre-treated RNA was reverse transcribed into cDNA for subsequent qRT-PCR detection. The primers used are listed in Supplemental Table S2.

### Northern blot

Northern Blot was performed as previously described (Edmonds et al. 1991). Briefly, 6 μg total RNA was separated by electrophoresis (100 V, 2 h) on a 15% urea PAGE, followed by immediate transfer onto a nylon membrane (Thermo Fisher, USA) (30 V, 2 h). Those processes were performed on ice. After drying the nylon membrane, it was UV cross-linked for 3 min on each side with an energy of 2400. The nylon membrane was then incubated with Buffer A at 37 °C for 30 min, followed by overnight incubation with the 3’-DIG–labeled probes (Sangon Biotech, China) at 37 °C. The membrane was first washed with Low Stringent Buffer, High Stringent Buffer and Washing Buffer at 37 °C. After the washes, the membrane was blocked with Blocking Buffer at room temperature for 3 h, and then incubated with DIG antibody (Sigma, USA) at room temperature for 30 min. The membrane was then washed with DIG Washing Buffer. Finally, the membrane was incubated with the luminescent substrate CSPD (Roche, Switzerland) at 37 °C in the dark for 20 min, and the luminescent signal was visualized using an imaging system. The probes and the buffers used are listed in Supplementary file 1.

### tiRNA interference and overexpression

Transfections of tiRNA inhibitor were performed using Lipofectamine® RNAiMAX Reagent (Thermo Fisher, USA) manufacturer’s protocols. Transfections of tiRNA mimics and endogenous tiRNA were performed using Lipofectamine® 3000 Transfection kit (Thermo Fisher, USA) manufacturer’s protocols. The inhibitors and mimics were synthesized by Sangon, and the sequences are provided in Supplementary file 1.

### Extraction for endogenous tiRNA

6 × 10^7^ cells were washed twice with 1 × HBSS and collected. The cells were centrifuged at 4 ℃ for 3 min at 1000 g. 250 μL of ANG digestion buffer (5 mM MgCl_2_, 0.5% NP-40, 1 × PBS pH7.4) was added to the cells, followed by centrifugation at 4 ℃ for 30 s at 1500 g to collect the supernatant. 100 nM angiogenin (ANG) (Novoprotein, China) was added to the supernatant and incubated at room temperature for 30 min. RNA was extracted using Trizol. Then the RNA was separated by electrophoresis (100 V, 2 h) on a 7.5% urea PAGE. The gel was transferred to a 3 × SYBR GelRed solution (Solarbio, China) and incubated at room temperature for 1–1.5 h. The gel containing tiRNA fragments was cut under a UV lamp. 1 × Elution buffer (20 mM Tris–HCl pH7.4, 250 nM CH_3_COONa, 1 mM EDTA, 0.25% SDS) was added to the gel and incubated at 65 ℃ for 15 min, followed by freezing at −80 ℃ for 15 min, and another incubation at 65 ℃ for 15 min. The supernatant was then collected and RNA was extracted using Trizol. 500 pmol biotinylated oligo probe (Ribiotech,China) and 950 μL of TBS buffer (0.05% NP-40, 200 U RNase Inhibitor) were added to the RNA and incubated at room temperature for 1 h. Streptavidin magnetic beads (Thermo Fisher, USA) washed three times with TBS buffer, were added and incubated at room temperature for 1 h. The magnetic beads were washed three times with TBS buffer for 5 min each time, and then Trizol was added to purify the RNA-probe complex. After digestion with DnaseI, the Trizol was added to extract RNA. The RNA was incubated at 4 ℃ for 2 h with m^7^G antibody (RNA: m^7^G antibody = 1:10) and 500 μL of 1 × IPP buffer (2 mM Tris–HCl pH7.4, 30 mM NaCl, 0.02% NP-40), followed by overnight incubation at 4 ℃ with Pierce™ Protein A/G Magnetic Beads (Thermo Fisher, USA) washed with 1 × IPP buffer. After washing the beads three times with 1 × IPP buffer, Trizol was added to extract the RNA, resulting in endogenous m^7^G 3' tiRNA.

### Statistical analysis

The data of 3 independent experiments were statistically analyzed (all with mean ± standard deviation). The software Graphpadprism8 (GraphPad, CA, USA) and SPSS 16.0 (IBM, Armonk, NY, USA) was used to analyze and plot the experimental data. The statistical methods were adjusted according to the type of experiment. Inspection level of α = 0.05, *P < *0.05, considered statistically significant, this paper identifies the NC: P > 0.05, * *P < *0.05, ** *P < *0.01, *** *P < *0.001, ****P < 0.0001.

## Results

### METTL1 promotes the formation of SGs induced by cadmium

**Fig. 1:**
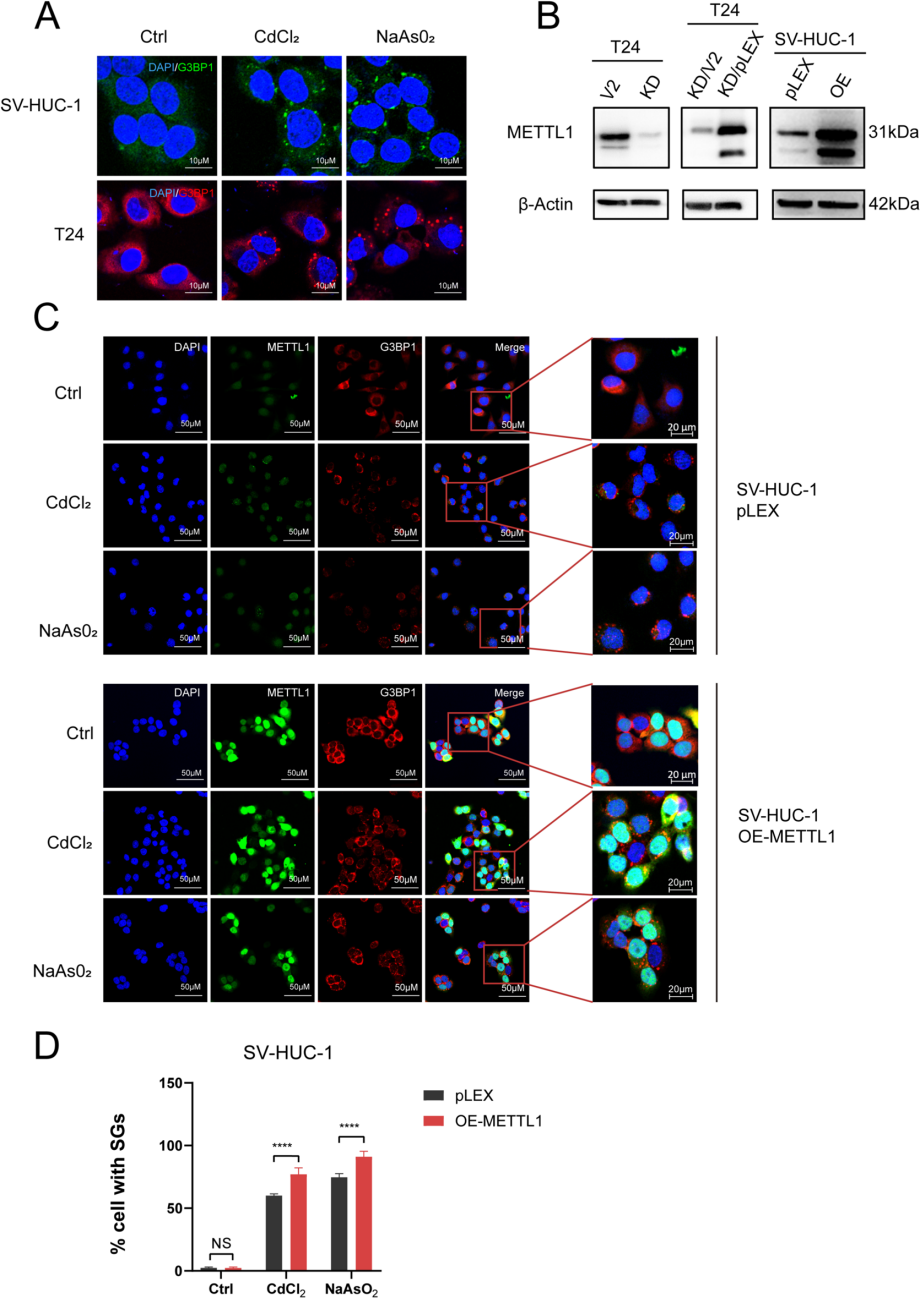
Overexpression of METTL1 upregulated the formation of SGs induced by cadmium. **A** Immunofluorescence (IF) assay was performed when T24 and SV-HUC-1 cells were exposed to CdCl_2_ and NaAsO_2_ (300 μM; 1 h) and stained for the SGs marker G3BP1. **B** T24 cell lines with CRISPR/Cas9-based knockdown METTL1 and SV-HUC-1 cell lines with LentiORF pLEX-MCS-based overexpression METTL1. Cells transfected with empty vector (T24-V2 and SV-HUC-1-pLEX) were used as control. **C** Compared to SV-HUC-1, IF assay showed higher expression of METTL1 and G3BP1 in METTL1-overexpressed SV-HUC-1 cells when exposed to CdCl_2_ and NaAsO_2_ (300 μM; 1 h). **D** The percentage of cells positive for SGs was quantified (= Number of cells with SGs/Total number of cells * 100%). Error bars indicate SD. The data are representative of three independent experiments

**Fig. 2:**
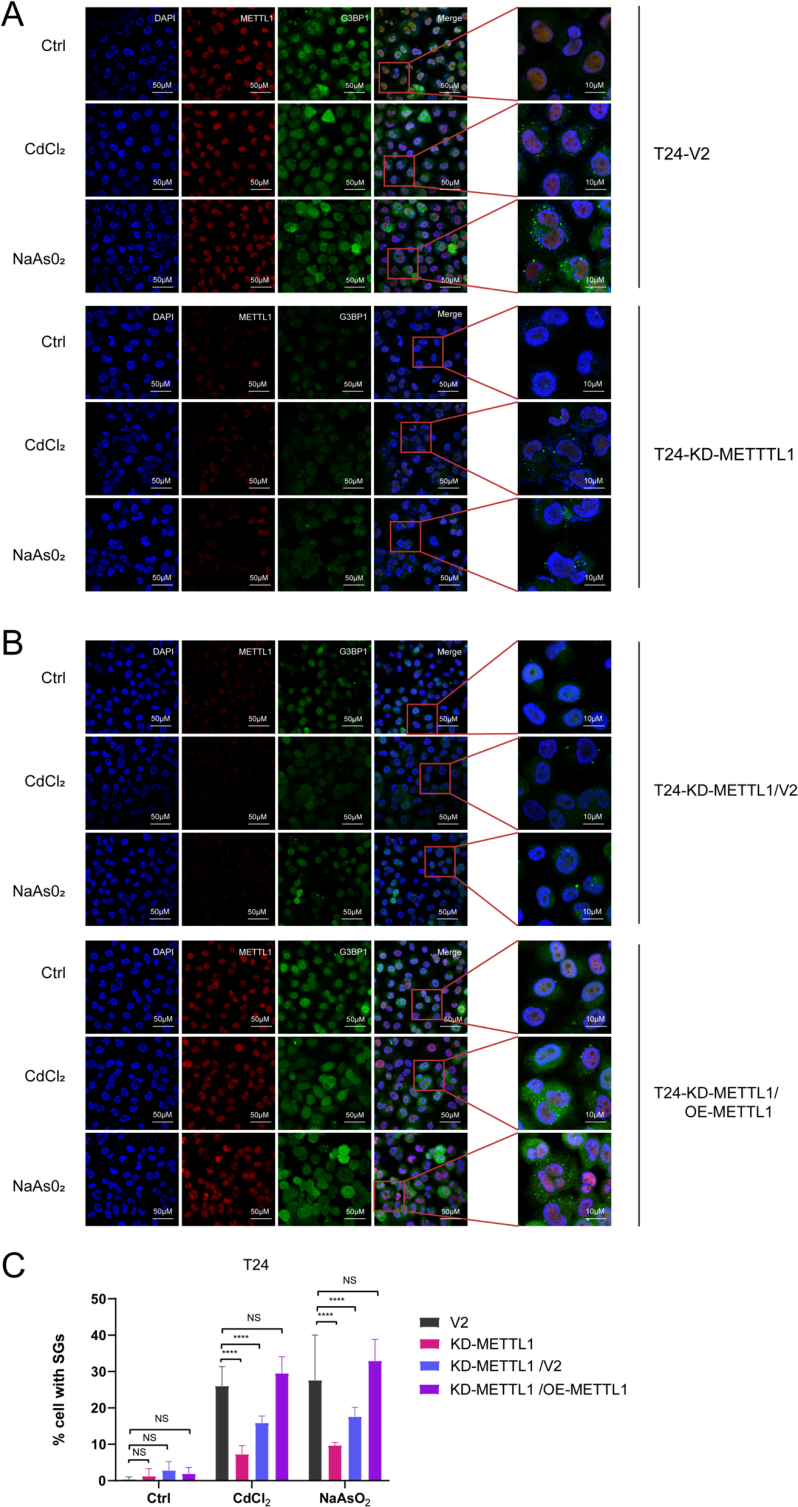
Restoring METTL1 expression alleviates the inefficiency of SG formation. **A-B** Immunofluorescence assays of T24-V2, T24-KD-METTL1, T24-KD-METTL1/V2, T24-KD-METTL1/OE-METTL1 cells after exposure to CdCl_2_ and NaAsO_2_ (300 μM; 1 h) using antibodies against METTL1 and G3BP1. **C** Statistical analysis of the proportion of SGs-positive T24 cells. The results were obtained from three separate experiments

To investigate the effect of METTL1 on the acute stress response in urothelial cells, we utilized the normal uroepithelial SV-HUC-1 cells expressed the low METTL1, and the bladder cancer T24 cells, which exhibits the high expression level of METTL1. We exposed SV-HUC-1 cells and T24 cells to a specific oxidative stressor—CdCl₂ (with NaAsO₂ as a positive control) and found that the cellular stress response was robust, leading to the formation of SGs (Fig. 1A). Due to high endogenous expression of METTL1 in T24 cells and low endogenous expression of METTL1 in SV-HUC-1 cells, we knocked down METTL1 in T24 cells using the CRISPR/Cas9 technology, and overexpressed METTL1 in SV-HUC-1 cells. Western blot confirmed the knockdown or overexpression of METTL1 cell lines (Fig. 1B), namely METTL1-knockdowned T24 cells (T24-KD-METTL1) and METTL1-overexpressed SV-HUC-1 cells (SV-HUC-1-OE-METTL1). The corresponding cells transfected with the control vector are T24-V2 and SV-HUC-1-pLEX, respectively. Additionally, we transfected T24-KD-METTL1 cells with a blank vector and a METTL1 overexpression plasmid, generating T24-KD-METTL1/V2 and T24-KD-METTL1/OE-METTL1 cells one by one (Fig. 1B). Then, we explored the optimal concentrations and time points for CdCl_2_ and NaAsO_2_ to induce the formation of SGs in cells, and determined that the most abundant stress-inducible SGs were observed in SV-HUC-1 and T24 cells treated with 300 μM CdCl_2_ and NaAsO_2_ for 1 h. (Fig. S1A-D, Supplementary file 1). Subsequently, we stimulated T24 V2, T24-KD-METTL1, SV-HUC-1 pLEX, and SV-HUC-1 OE-METTL1 cells with 300 µM CdCl₂ and NaAsO₂ individually for 1 h, and visualized the formation of SGs by immunofluorescence using G3BP1 and TIA-1 as markers. The results confirmed that the knockdown of METTL1 significantly reduced the percentage of cells with SGs compared to the control T24-V2 cells (Fig. 2A-C, Fig. S2B, D, Supplementary file 1), while overexpression of METTL1 observably increased the percentage of cells with SGs (Fig. 1C-D, Fig. S2A, C, Supplementary file 1). Moreover, METTL1-knockdowned T24 cells were transfected with the METTL1 overexpressed plasmid to restore METTL1 expression, and the effect of METTL1 knockdown on SGs formation was largely eliminated upon restoration of METTL1 expression (Fig. 2A-B). These results confirmed that METTL1 significantly influenced the formation of SGs induced by cadmium.

### METTL1 upregulates G3BP1 protein expression

**Fig. 3:**
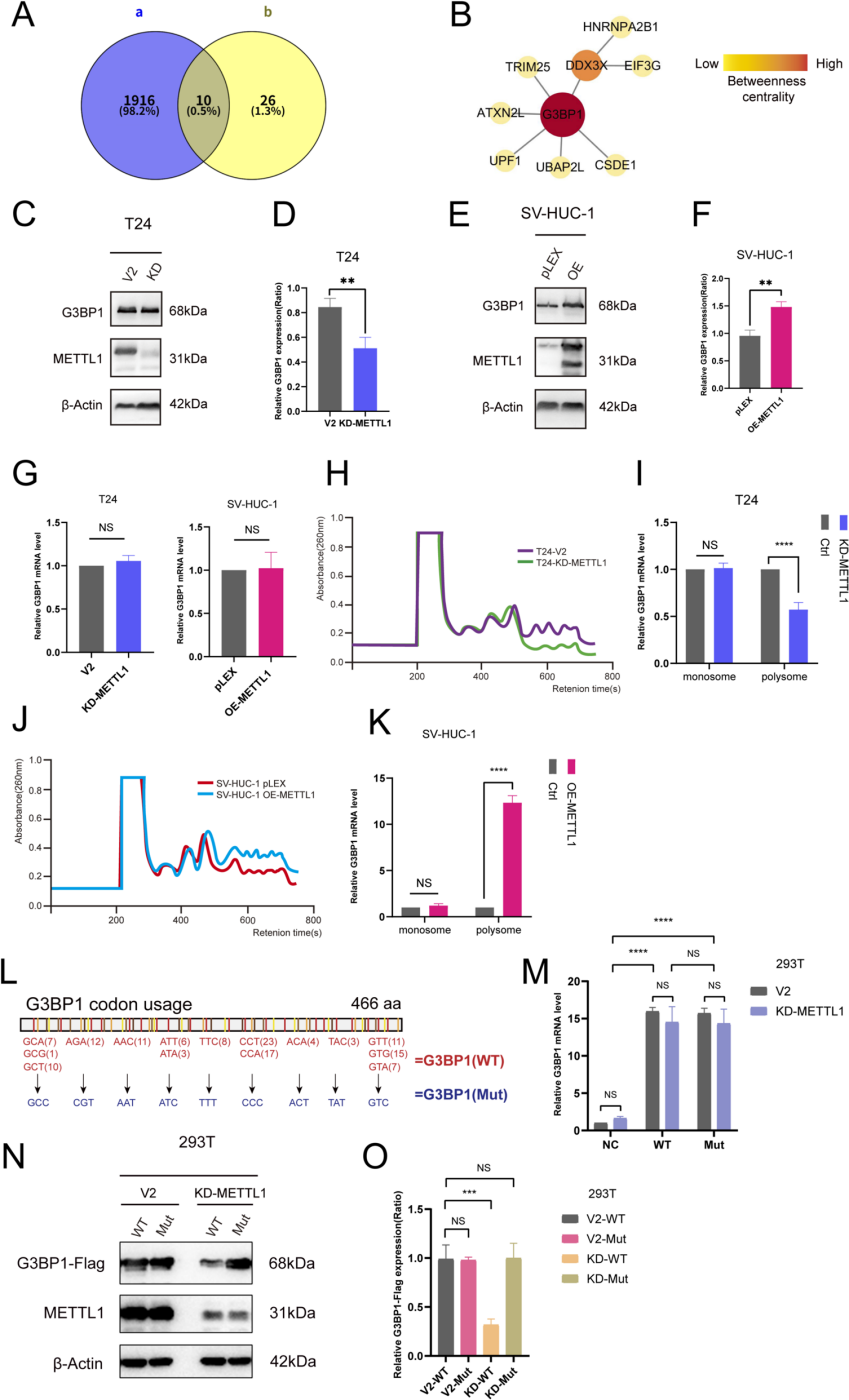
METTL1 affects the translation of G3BP1 rather than transcription. **A **The Venn analysis of differentially expressed genes (DEGs) of the Ribo-seq results of T24-KD-METTL1 cells compared to T24 V2 cells(a) and SGs core proteins under arsenate stress(b). **B** The protein–protein interaction network of DEGs. **C-F** Western blot analyzed the G3BP1 level of T24-V2, T24-KD-METTL1(C), SV-HUC-1-pLEX and SV-HUC-1-OE-METTL1 cells(E), respectively. Bar-chart reflecting expression levels of G3BP1 after knockdown(D)/overexpression(F) of METTL1 in figure B. **G** qRT-PCR confirmed that the knockdown or overexpression of METTL1 had no significant effect on G3BP1 mRNA. **H–K** Polysome profile of T24-KD-METTL1/T24 V2(H) and SV-HUC-1 OE METTL1/SV-HUC-1 pLEX(J). Bar chart depicted G3BP1 mRNA level between T24-KD-METTL1/T24 V2(I) and SV-HUC-1 OE METTL1/SV-HUC-1 pLEX(K) **L** From 5' to 3', diagram showing the G3BP1 mRNA codons bound to m^7^G-tRNA and corresponding site-mutation plasmid construction strategies. **M** qRT-PCR illustrated the transfection efficiency of G3BP1 wild-type (WT) and mutant (Mut) plasmids in 293T V2 and 293T-KD-METTL1 cells. **N** Western blotting demonstrated the rescue effect of G3BP1 from G3BP1-Mut plasmid transfected 293T cells, detected by anti-Flag antibody. **O** Bar-chart reflecting expression levels of G3BP1-Flag in figure N. The results were obtained from three separate experiments

To further investigate how METTL1 influences the formation of SGs, we screened for representative ribosome-protected transcripts which function as key participants of SGs assembly by using our previous ribosome profiling sequencing (Ribo-seq) data on T24-KD-METTL1 cells (Ying et al. 2021). We identified 10 SG core proteins whose translation was significantly affected by METTL1 knockdown in T24 cells (Fig. 3A, Supplementary file 2). We conducted a protein–protein interaction analysis of these 10 proteins using STRING (set at high confidence). Nine of the proteins exhibited direct interactions, with G3BP1 positioned at the most central location. Therefore, we focused our attention primarily on G3BP1 (Fig. 3B). To verify METTL1 correlates with G3BP1 expression, we examined the expression levels of G3BP1 protein in T24-V2, T24-KD-METTL1, SV-HUC-1 pLEX, and SV-HUC-1 OE-METTL1 cells. The result showed that the protein expression of G3BP1 is reduced in T24 cells with METTL1 downregulation, while the protein expression of G3BP1 is increased in SV-HUC-1 cells with METTL1 overexpression (Fig. 3C-F), with no significant changes at the mRNA level (Fig. 3G). Immunofluorescence also confirmed that METTL1 overexpression promotes G3BP1 protein expression, while METTL1 knockdown has the opposite effect (Fig. 1C, 2A). Considering that Ribo-seq identified a downregulation of G3BP1 at the translational level after METTL1 knockdown, we speculated that METTL1 regulates the translation of G3BP1 rather than its transcription.

### METTL1 regulate G3BP1 protein translation by mediating tRNA m^7^G modification

To further validate Ribo-seq and demonstrate the impact of METTL1 on the translational level of G3BP1, we first performed mRNA polysome analysis using sucrose gradient fractionation, which enables us to detect G3BP1 mRNA abundance and its translational efficiency. The knockdown of METTL1 resulted in a significant reduction in the polyribosome peak, indicating a potential disruption in the translation process. Conversely, the polyribosome peak was significantly increased following the overexpression of METTL1, suggesting that METTL1 plays a crucial role in maintaining normal polyribosome levels and, by extension, the efficiency of translation in BC cells (Fig. 3H, J). The results of qRT-PCR indicate that the absence of METTL1 leads to global inhibition of G3BP1 protein translation, while its overexpression promotes the translation of G3BP1 (Fig. 3I, K), thereby validating the results of the Ribo-seq sequencing. Subsequently, we conducted a protein synthesis inhibitor cycloheximide (CHX) chase assay to ascertain whether METTL1 affects G3BP1 degradation. We treated T24 and SV-HUC-1 cells with CHX for 0 h, 2 h, 4 h, 6 h, and 8 h. Whole cell proteins were examined by western blotting that the expression of G3BP1 protein in both the control group and the METTL1-KD/OE group gradually decreased with the prolongation of CHX exposure (Fig. S3A-D, Supplementary file 1). This indicates that there are no notable differences in half-life of G3BP1 between the two groups, suggesting that METTL1 does not exert a significant influence on the stability of G3BP1.

The RNA methyltransferase METTL1 is linked to the catalysis of N (7)-methylguanine at position 46 (m^7^G_46_) in a large subset of tRNAs(Orellana et al. 2021). To determine whether METTL1 regulates G3BP1 protein translation by mediating tRNA m^7^G modification, we analyzed the codon usage frequency of the G3BP1 mRNA. We specifically mutated wild-type codons which decoded by m^7^G-tRNA and replaced them with codons decoded by non-m^7^G-tRNA isoacceptors. This approach led to the construction of overexpression plasmid for G3BP1 mutant synonymously mutated all m^7^G-tRNA decoding codons (Fig. 3L). Then, equal amounts of wild-type and mutant G3BP1 plasmids were transiently transfected into 293T-V2 or 293T-KD-METTL1 cells, and the results of qRT-PCR confirmed the similar efficiency of transfection (Fig. 3M). Western blotting showed that the protein level of G3BP1 in wild-type-transfected 293T control cells was higher than that in cells transfected with the mutant. However, upon knockdown of METTL1 expression, there were significant increased protein expression of G3BP1 in cells transfected with the mutant, compared with the wild-type transfection (Fig. 3N, O). In conclusion, we have directly confirmed that G3BP1 expression is regulated by tRNAs with m^7^G modification whose writer is precisely METTL1.

### METTL1 regulates the expression of tiRNAs in SG core RNA

SGs are membraneless organelles with a dynamic shell and stable core structure made up of RNAs and proteins (Brocato and Costa 2013). The SG core can be purified, followed by identification of the core protein and its transcriptome. Previously, we found that during the transformation of urothelial cells induced by the CdCl_2_, the level of tRNA m^7^G modification increased with the extension of CdCl_2_ treatment time (Ying et al. 2024). Considering the catalytic role of METTL1 in m^7^G methylation modification of tRNA, we were able to extract SGs core RNAs to investigate the effect of METTL1-mediated m^7^G modification on SG formation. Therefore, we generated engineered SV-HUC-1 cell lines expressing the G3BP1-EGFP fusion protein, with (w/) or without (w/o) the overexpression of METTL1 (Fig. S4B-C, Supplementary file 1), and used an extraction method described by Khong et al. (2018) for the isolation and purification of SG core RNAs (Fig. S4A, Supplementary file 1).

**Fig. 4:**
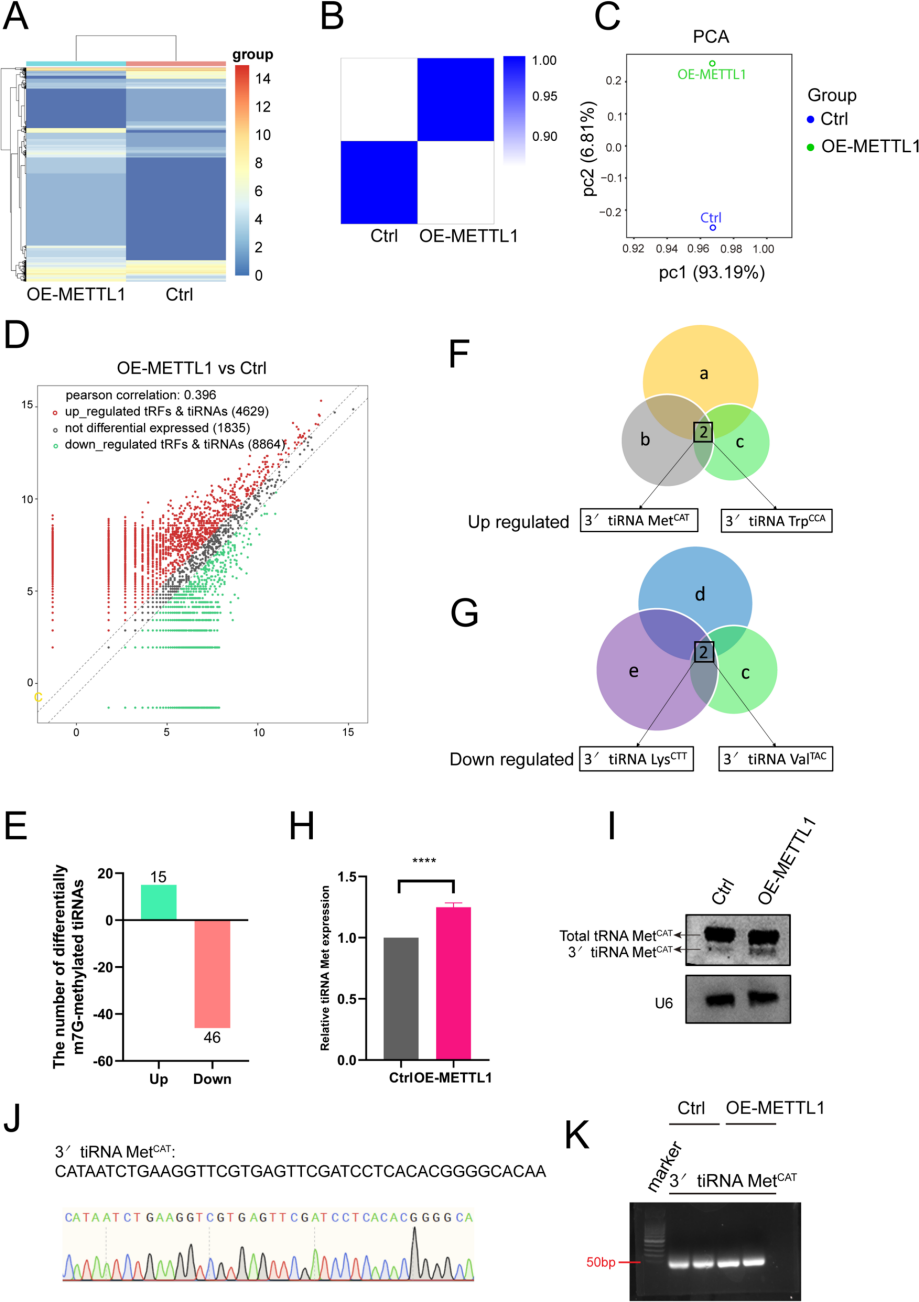
Screening and validation of SG core tRFs & tiRNAs. **A-C** The unsupervised hierarchical heatmap of correlation coefficient (A), clustering heatmap (B) and PCA analysis (C) between SC-HUC-1 pLEX and SV-HUC-1 OE-METTL1 cells. **D** The volcano plot quantified the number of tRNAs with differential expression that was either up- or down-regulated between SC-HUC-1 pLEX and SV-HUC-1 OE-METTL1 cells. **E** Bar chart shows the intersection of the number of tsRNAs with the same trend of differential expression as m^7^G-tsRNAs (from Arraystar Human m^7^G small-RNA-modified microarrays) and tsRNAs from SG core tsRNA sequencing. **F-G** Venn diagram for SG core tsRNAs, m^7^G tsRNAs, and tsRNAs with m^7^G modification motifs in BC. a: Upregulated differential tsRNAs in SV-HUC-1 cells overexpressing METTL1. b: Downregulated differential m^7^G tsRNAs in T24-KD-METTL1 cells. c: tsRNAs with m^7^G modification motifs. d: Downregulated differential tsRNAs in SV-HUC-1 cells overexpressing METTL1. e: Upregulated differential m^7^G tsRNAs in T24-KD-METTL1 cells. **H** qRT-PCR quantified the expression level of tiRM in METTL1-overexpressed SV-HUC-1 and control SV-HUC-1 cells with a representative bar chart. **I** Northern blotting also validated the expression of tiRM in METTL1-overexpressed SV-HUC-1 cells. **J** Sanger sequence of 3' tiRNA Met^CAT^, excluded amino acid arm CCA at the 3' end. **K** Agarose gel electrophoresis develop of 3' tiRNA Met^CAT^ from qRT-PCR product

We performed tRFs & tiRNAs sequencing on the extracted SG core RNAs to distinguish differential tRFs or tiRNAs expression between the METTL1-overexpressed SV-HUC-1 cells and the control SV-HUC-1 cells (Fig. 4A-C). We compared the detected tsRNAs (Fig. 4D, Supplementary file 3) with m^7^G- tsRNAs by the Arraystar Human m^7^G small RNA-modified microarrays in METTL1 knockdown T24 cells and control cells (Ying et al. 2024) for Venn diagram analysis (Supplementary file 4). After excluding the non-significantly expressed tsRNAs, we identified 15 tsRNAs that were up-regulated after METTL1 overexpression and down-regulated after METTL1 knockdown (fold change (FC) ≥ 2 or < 0.5 respectively, *p*-value < 0.05). Additionally, we found 46 tsRNAs that were down-regulated after overexpression of METTL1 and up-regulated after knockdown of METTL1 (Fig. 4E, Supplementary file 5).

Given that stress-activated tRNA cleavage is rooted in the anticodon loop into 3' tiRNAs and 5' tiRNAs, we focused on 3' tiRNAs that also harbor m^7^G motifs identified in our previous research in the bladder cancer cell line T24 (Ying et al. 2021). Subsequently, we performed a three-way Venn diagram intersection analysis involving tsRNA sequencing, m^7^G small-RNA modification microarrays, and tsRNAs with m^7^G-modified motifs in BC. From this analysis, we identified two differentially upregulated tiRNAs (3' tiRNA Met^CAT^ and 3' tiRNA Trp^CCA^) (Fig. 4F) and two downregulated tiRNAs(3' tiRNA Lys^CTT^ and 3' tiRNA Val^TAC^) (Fig. 4G, Supplementary file 6). These tiRNAs were m^7^G-modified 3' tiRNAs, which were the primary focus of our analysis based on their relevance to stress responses. Due to stress-induced increases in tiRNAs, we selected the two tiRNAs that were upregulated upon overexpression of METTL1 and downregulated upon knockdown of METTL1.

### m^7^G-3' tiRNA Met^CAT^ promotes SGs formation

To validate the screening results from the sequencing data, we detected the 3' tiRNA Met^CAT^ (tiRM) expression in METTL1-overexpressed SV-HUC-1 cells. qRT-PCR and Northern blotting showed an upregulated expression compared to non-METTL1-overexpressed cells (Fig. 4H, I). Subsequently, we determined the sequence of tiRM using Sanger sequencing (Fig. 4J). The obtained tiRM sequence was consistence with our screening results. Agarose gel electrophoresis also developed a single lane of tiRM, indicating that the validating results were unique and not interfered by other fragments (Fig. 4K). Therefore, tiRM may serve as an intermediary between METTL1 and SGs formation.

**Fig. 5:**
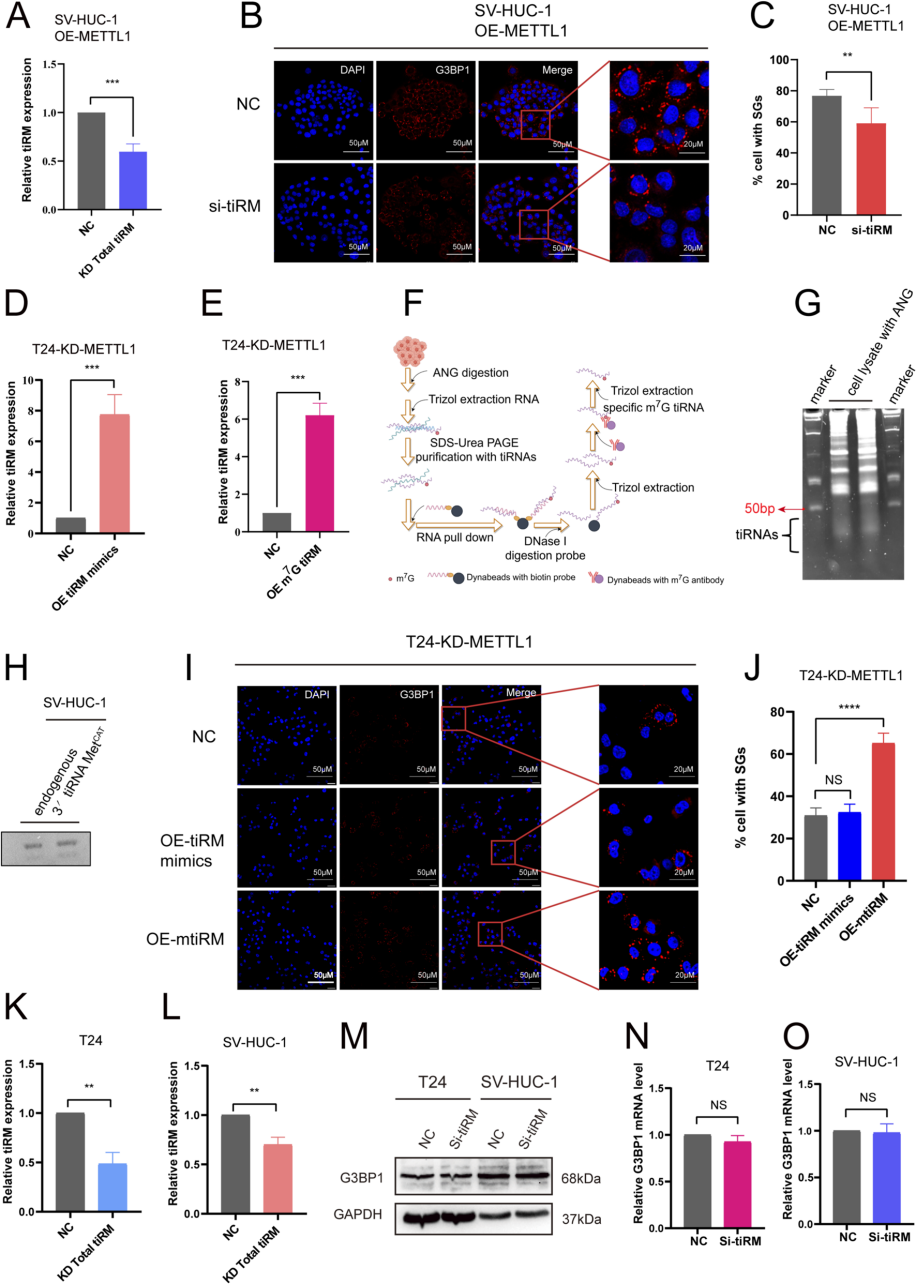
Effect of m^7^G modification on 3' tiRNA Met^CAT^ in SGs formation.** A** After transient transfection of tiRM inhibitor into SV-HUC-1 OE-METTL1 cells, the expression levels of tiRM were measured in transfected cells compared to cells transfected with negative control (NC). **B, C** METTL1-overexpressed SV-HUC-1 cells transfected tiRM inhibitor and NC were exposed to CdCl_2_ (300 μM, 1 h). G3BP1 is the marker of SGs. **D, E** tiRM mimics and mtiRM were transfected into T24-KD-METTL1 cells, and the efficiency of overexpression was detected by qRT-PCR. **F** Workflow for the extraction of mtiRM (Graphing website: https://www.figdraw.com/). **G** The image shows the develop results of SDS-PAGE separation of tiRNAs. **H** mtiRM was verified by Northern Blot assay. **I, J** T24-KD-METTL1 transfected with tiRM mimics, mtiRM and NC were exposed to CdCl_2_ (300 μM, 1 h) (I), and the percentage of positive cells for SGs was quantified (J). **K, L** The knockdown efficiency of total tiRM was detected by qRT-PCR after transfecting tiRM inhibitor into SV-HUC-1(K) and T24(L) cells. **M–O** After knocking down the expression of total tiRM in SV-HUC-1 and T24 cells, the level of G3BP1 protein(M) and mRNA (N, O) was not significantly changed

Then we explore the hidden impact of tiRM on the formation of SGs, we first synthesized inhibitor to inhibit tiRM in METTL1-overexpressed SV-HUC-1 cells (Fig. 5A). The IF analysis demonstrated a notable decline in the proportion of cells exhibiting positive staining for SGs following the disruption of tiRM expression (Fig. 5B, C, Fig. S5A, B, Supplementary file 1), suggesting an interwind relationship between tiRM and SGs formation. Hence, we transfected synthetic tiRM mimics into T24-KD-METTL1 cells to increase the exogenous level of tiRM (Fig. 5D). The data indicated that simply increasing the expression of the tiRM sequence did not result in a more pronounced difference in the number of SG-positive cells when compared to the NC group (Fig. 5I, J, Fig. S5A, B, Supplementary file 1). Since the synthesized tiRM mimics lack the METTL1-mediated m^7^G modification present in endogenous tiRM, we postulate that the m^7^G modification of endogenous tiRM may affect the formation of SGs.

To demonstrate that endogenous m^7^G-tiRM affects SGs formation, we designed an improved method for isolating m^7^G-3' tiRNA Met^CAT^ (mtiRM) by modifying the methods previously described for isolating specific tiRNA (Fig. 5F, G) (Akiyama et al. 2020, 2021). The extracted mtiRM was validated by Northern blot (Fig. 5H). The extracted mtiRM was later transfected into T24-KD-METTL1 cells (Fig. 5E). IF analysis revealed a significant increase in the percentage of SG-positive cells following mtiRM overexpression (Fig. 5I, J, Fig. S5C, D, Supplementary file 1). Considering that METTL1 affects the translation of G3BP1 protein, we hypothesized whether m^7^G-modified tiRM, which are also regulated by METTL1, directly influence the expression of G3BP1. To test this, we transfected a tiRM inhibitor into SV-HUC-1 and T24 cells to inhibit total tiRM level (Fig. 5K, L). Subsequently, we examined the mRNA and protein levels of G3BP1 and found no significant change (Fig. 5M-O). Therefore, we are convinced that mtiRM does not affect the expression of G3BP1.In conclusion, our findings indicate that METTL1 plays a role in promoting SGs formation by modulating the m^7^G modification of 3' tiRNA Met^CAT^.

## Discussion

**Fig. 6:**
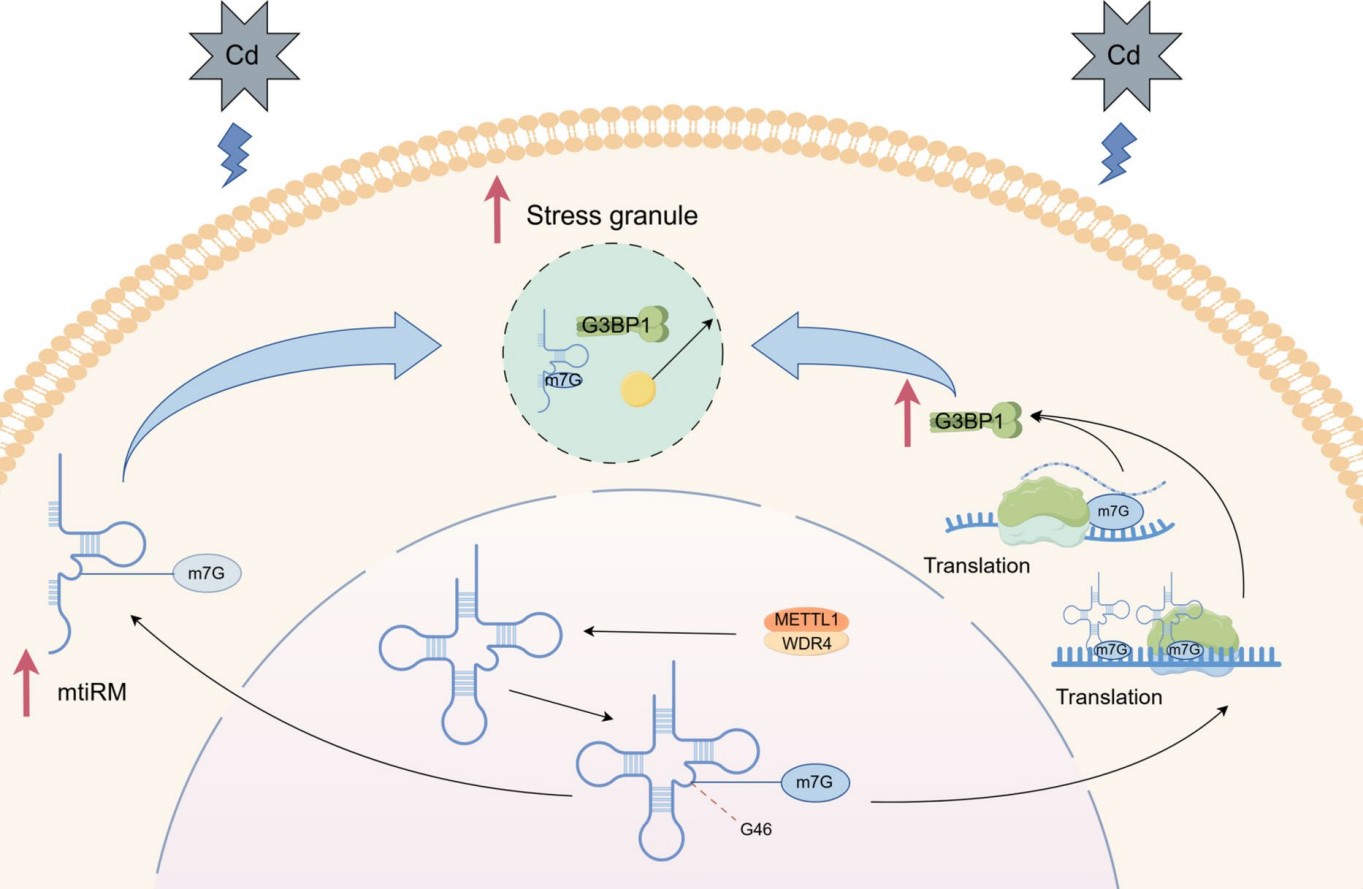
Summary of the regulation of stress granule assembly by METTL1 via G3BP1 and mtiRM

Our previous study unveiled the role and molecular mechanism of a novel m^7^G-modified tsRNA, m^7^G-3′ tiRNA Lys^TTT^ (mtiRL), in the malignant progression of bladder cancer. mtiRL exhibits high expression levels in SV-HUC-1 cells at different stages of CdCl_2_-induced malignant transformation, as well as in a rat model of bladder cancer subjected to CdCl_2_ treatment, promoting the occurrence and malignant progression of BC. However, the mechanism of effect of m^7^G-modified tsRNAs in the early stages of cadmium exposure has not yet been elucidated. In this study, we confirmed that in the early stages of cadmium exposure, METTL1 promotes the assembly of SGs in BC cells. By combining Ribo-seq analysis with METTL1 knockdown relative to the control group, we found that the translation level of the core protein of SGs, G3BP1, was downregulated. Subsequently, we verified that METTL1 regulates the translation level of G3BP1 protein by mediating m^7^G modification and affects the formation of SGs. Furthermore, we found that m^7^G-3' tiRNA Met^CAT^ (mtiRM) affects the assembly of SGs through core small RNA sequencing of SGs and m^7^G small RNA modification microarray, confirming that METTL1 also regulates the assembly of SGs by affecting the abundance of mtiRM. Overall, these results demonstrate the bidirectional function and mechanism of METTL1 in the assembly of SGs under stress (Fig. 6).

In recent years, heavy metals have become a prevalent environmental pollutant due to extensive industrial activities, with cadmium (Cd) emerging as a particularly significant concern (Balali-Mood et al. 2021). Alongside other non-essential heavy metals such as arsenic (As), chromium (Cr), lead (Pb), nickel (Ni), and mercury (Hg), cadmium's non-degradable nature and long half-life make it a persistent threat. These metals can accumulate in the environment and human organs, triggering pathological changes in tissues and organs (Manfo et al. 2012). Additionally, cadmium exposure is associated with increased production of reactive oxygen species (ROS) and oxidative stress within cells, which weakens antioxidant defenses and leads to cellular damage (Coradduzza et al. 2024). Notably, cadmium, along with As, Cr, and Ni, is classified as a Class I carcinogen, capable of inducing oxidative stress, disrupting DNA synthesis and repair, and causing abnormal DNA methylation (Boffetta 1993; Kim et al. 2015). These mechanisms are implicated in various cancers, including lung, renal, liver, bladder, pancreatic, and gastric cancers (García-Esquinas et al. 2014; Kim and Sharma 2004). Oxidative stress is considered an important mechanism for the carcinogenicity of cadmium (Bhardwaj et al. 2021). Through immunofluorescence experiments, we found that in the early stages of exposure to CdCl_2_, METTL1 can promote the production of stress granules (SGs) in cells, which further confirms the important role of METTL1 in the assembly of SGs.

SGs function as a protective mechanism in cellular stress responses by aggregating stalled translation machinery, including mRNAs, ribosomal components, translation initiation factors, and RNA-binding proteins, thereby facilitating cell survival under adverse conditions (Shil et al. 2023). Acting as temporary storage sites for mRNAs and proteins, SGs modulate the expression of specific proteins (e.g., Hsp70) and mRNAs, aiding in the rapid restoration of cellular functions under stress (Moser and Fritzler 2010). Furthermore, the formation of SGs is interconnected with multiple signaling pathways, which can regulate or alter cell growth, survival, or metabolism, and may even induce apoptosis (Marcelo et al. 2021). SGs have been implicated in various diseases, including neurodegenerative disorders such as Alzheimer's disease (Jo et al. 2020). In cancer, SGs exhibit a dual role. On one hand, they enable cancer cells to survive in harsh microenvironments. On the other hand, they can influence cancer cell invasion, metastasis, and drug resistance by modulating cellular signaling pathways and gene expression (Cheng et al. 2025).

To further explore how METTL1 affects the formation of SGs, we first considered the core proteins of SGs. The "core protein network" of SGs includes 36 proteins that participate in and highly influence the assembly of SGs. G3BP1 and G3BP2, which belong to the Ras-GTPase-activating protein-binding protein family, hold a central position in this network and determine the assembly of SGs under arsenate stress (Yang et al. 2020). Through ribosome profiling analysis, we found that the translation level of G3BP1 protein and the other 9 proteins level changed after METTL1 knockdown. In recent years, numerous studies have demonstrated the association of G3BP1 with various diseases, such as digestive system tumors, reproductive system tumors, urinary system tumors, and respiratory system tumors (Ge et al. 2022; Liu et al. 2022; Mukhopadhyay and Zhou 2022). Moreover, G3BP1 is highly expressed in cancer cells and mediates cancer cell proliferation, metabolism, and invasion through a series of oncogenic pathways (Zhang et al. 2019). Therefore, we chose G3BP1 as the subject of our next research. As for the other 9 genes, they occupy a secondary position in our PPI analysis network. In future research, we will continue to focus on these genes, but we will not delve into them in this study. After overexpressing/knocking down METTL1, the post-transcription level of G3BP1 protein in cells was significantly higher/lower than that of the control group, but there was no significant change in mRNA levels. Those results indicated that METTL1 affects the post-transcriptional level of G3BP1 protein rather than the transcription level. Subsequently, we verified the impact of METTL1 on the translation level of G3BP1 through polysome profiling, protein stability experiments, and mutating the m^7^G tRNA-recognition codon of G3BP1, and found that METTL1 promotes the expression of G3BP1 protein by affecting its translation, which is beneficial for the formation of SGs.

Due to the excessive proliferation driven by carcinogenesis, which requires a significant consumption of cellular resources, cancer cells are often under stress (Solimini et al. 2007; Urra et al. 2016). In order to reduce the energy expenditure of protein synthesis, the ribonuclease ANG is activated to cleave tRNAs, producing a large number of tiRNAs (Sarangdhar and Allam 2021a). The phosphorylation of the eukaryotic translation initiation factor eIF2α inhibits mRNA translation, and a large amount of mRNA aggregates and interacts with RNA-binding proteins, leading to liquid–liquid phase separation and the formation of SGs (Anderson and Kedersha 2009; Kedersha et al. 2013; Yang et al. 2020). During the formation of SGs, some microRNAs, translation initiation factors, large and small ribosomal subunit proteins, and a vast protein network are recruited (Elkordy et al. 2018; Khong et al. 2017), among which tiRNAs also promoting the assembly of SGs (Kedersha et al. 2013; Sarangdhar and Allam 2021b). Recent studies have found that METTL1 mediates the recruitment of m^7^G-modified mRNA within cells to SGs more readily under stress conditions (Zhao et al. 2023). Additionally, tRNA modifications affect the stability and production of tsRNAs, leading us to speculate that METTL1 may influence the formation of SGs by regulating tiRNAs.

Subsequently, we extracted core RNA from SGs and performed small RNA sequencing. After screening and validation, we discovered a novel m^7^G-modified tsRNA-3' tiRNA Met^CAT^ (mtiRM) whose expression level was upregulated in the METTL1 overexpression group. We then synthesized 3' tiRNA Met^CAT^ inhibitors and mimics and extracted endogenous 3' tiRNA Met^CAT^. IF experiments results revealed that interfering with tiRM inhibits the formation of SGs. Transient transfection of mimics into T24-KD-METTL1 cells did not significantly affect the formation of SGs, while overexpression of endogenous 3' tiRNA Met^CAT^ promoted SGs assembly. Based on these results, we concluded that METTL1 can promote the formation of SGs by mediating the m^7^G modification of 3' tiRNA Met^CAT^.

In conclusion, this study found that the methyltransferase METTL1 promotes the formation of SGs by regulating the translation of the SG core protein G3BP1 and mediating the m^7^G modification of 3' tiRNA Met^CAT^, providing new theoretical basis for the molecular mechanisms of bladder cancer induced by cadmium. However, the precise molecular mechanisms by which mtiRM promotes SGs assembly remain to be elucidated. Given that m^7^G modification is a positively charged modification, we hypothesize that m^7^G modification enhances the interactions between RNA molecules and increases the stability of tiRNA. These enhanced interactions and stability facilitate the aggregation and stabilization of RNA molecules within SGs, thereby promoting the formation and maintenance of SGs. Additionally, m^7^G-modified tiRNA can interact with various RNA-binding proteins, which play crucial roles in SG assembly. The signaling pathways involved in mtiRM function will also be a focus of our future research. We anticipate that these studies will provide a more comprehensive understanding of the role of m^7^G-modified tiRNA in the cellular stress response and lay the foundation for its potential therapeutic applications.

## Supplementary Information

Below is the link to the electronic supplementary material.

Supplementary file1 (DOCX 9187 KB)

Supplementary file2 (XLSX 2599 KB)

Supplementary file3 (XLSX 1759 KB)

Supplementary file4 (XLSX 255 KB)

Supplementary file5 (XLSX 1964 KB)

Supplementary file6 (XLSX 43 KB)

Supplementary file7 (PDF 137 KB)

## Data Availability

The SGs core RNA sequencing data are available via GEO at GSE232579.
