# Supplementary material for: METTL1 promotes cadmium-induced stress granules formation via enhancing translation of G3BP1 and expression of m7G- 3' tiRNA MetCAT
Source: Cell Biol Toxicol. 2025 Aug 5;41(1):124. doi: 10.1007/s10565-025-10072-0 (PMC12325392; doi:10.1007/s10565-025-10072-0)
Supplement: Supplementary file 1 — Supplementary file1 (DOCX 9187 KB) [file 10565_2025_10072_MOESM1_ESM.docx]

**Figure Lengend**

**
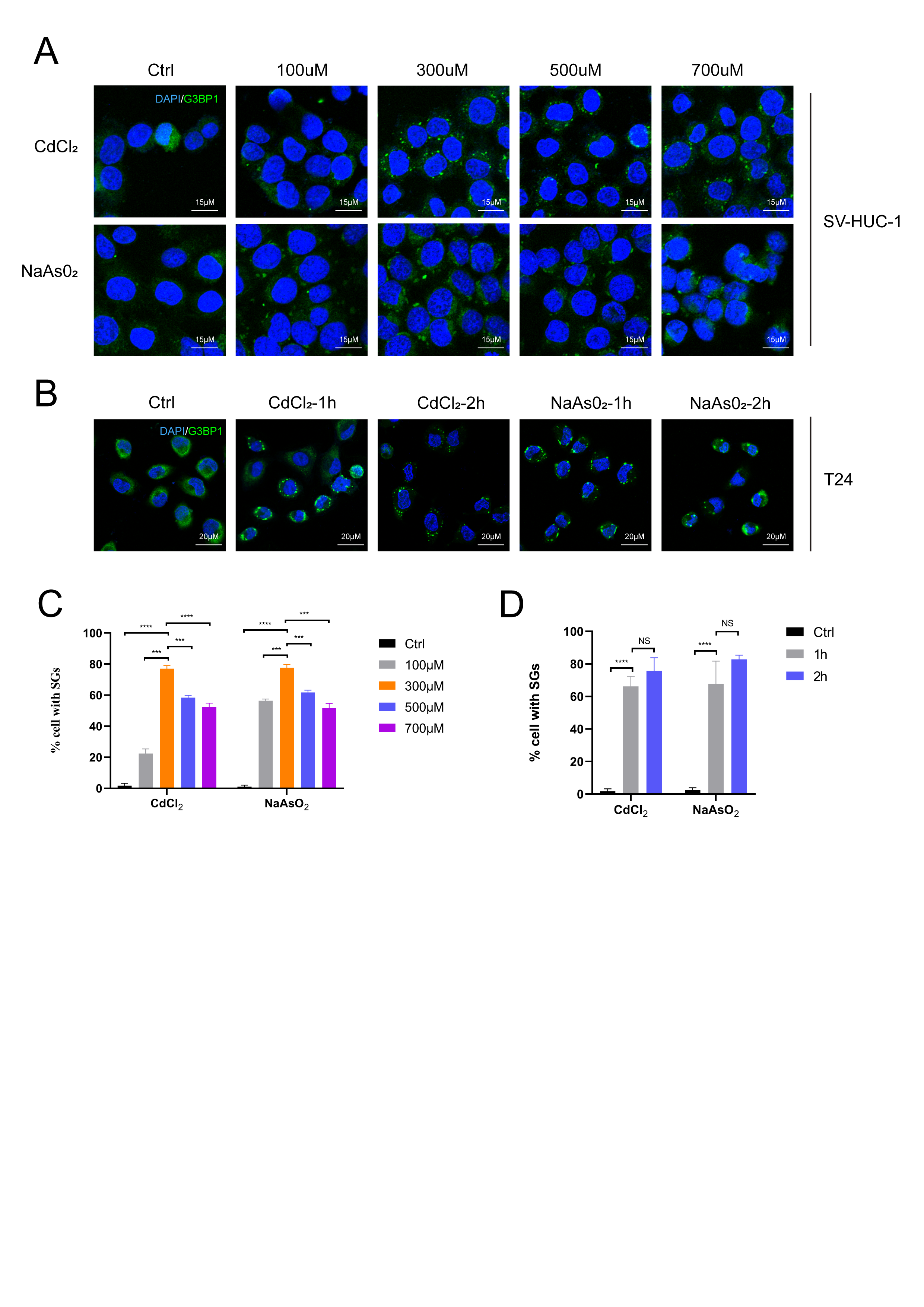
**

**Fig. S1** The best processing time and concentration for treating cells with CdCl_2_ and NaAsO_2_. **A** SV-HUC-1 cells were exposed to increasing concentrations of 100μM, 300μM, 500μM to 700μM CdCl_2_ or NaAsO_2_ for 1h. **B** T24 cells were exposed to 300μM CdCl_2_ or NaAsO_2_ for 1h or 2h. **C** Statistical Analysis of A. When the concentrations of CdCl_2_ and NaAsO_2_ are at 300 µM, the positive cell rate for the production of SGs is the highest. **D** Statistical Analysis of B. Treating cells with 300 µM CdCl_2_ or NaAsO_2_ for 1h results in the production of SGs that satisfy experimental requirements.


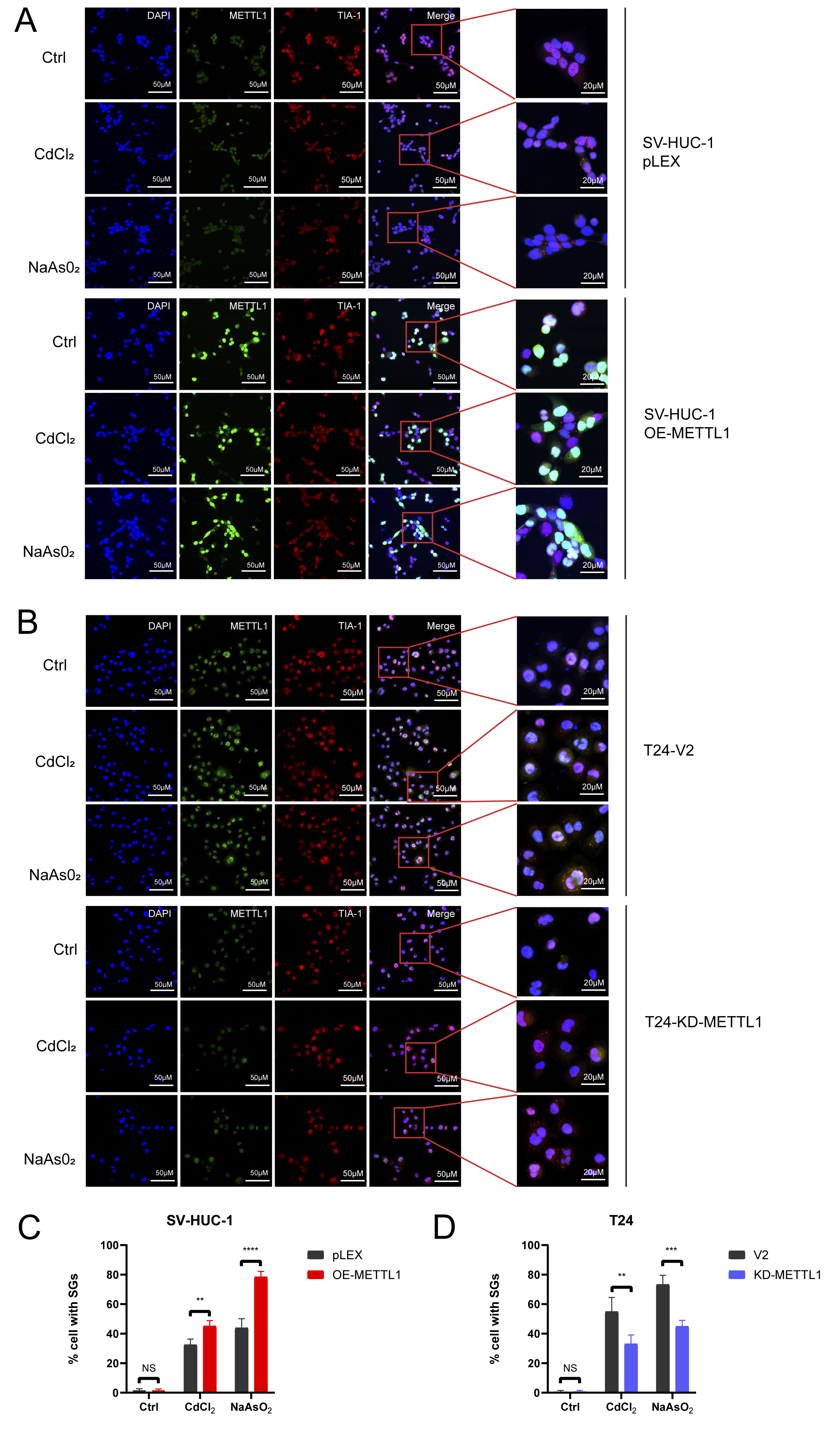


**Fig. S2 METTL1 affected the formation of SGs. A-B** IF assay was performed when cells SV-HUC-1 pLEX, SV-HUC-1 OE METTL1(A), T24 V2 and T24-KD-METTL1(B) were exposed to CdCl_2_ and NaAsO_2_ (300μM; 1 h) and stained for the SGs marker TIA-1. C-D Statistical analysis of the proportion of SGs-positive SV-HUC-1(C) and T24 cells(D). The data are representative of three independent experiments.

**
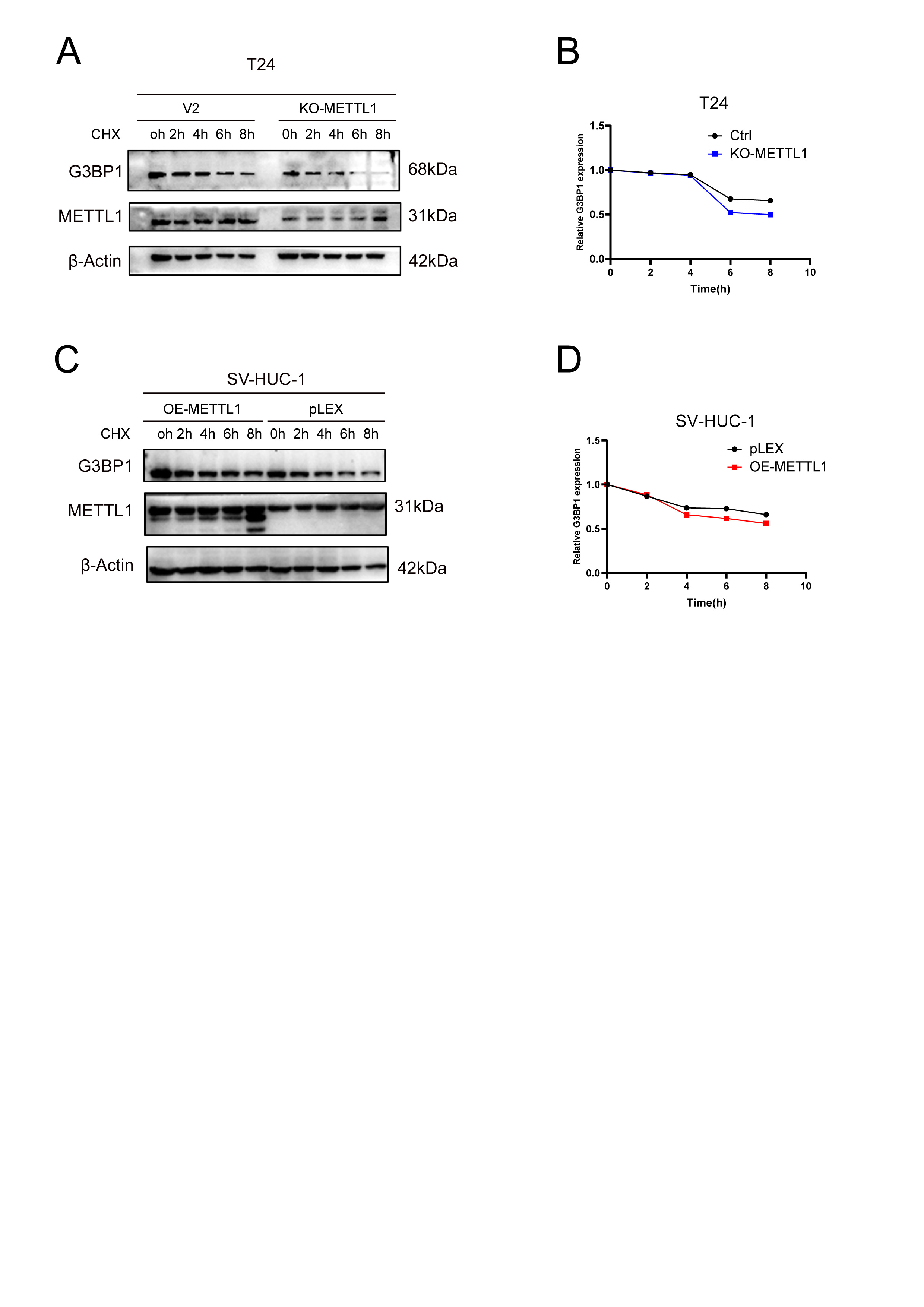
Fig. S3** The stability of G3BP1 protein is not affected by METTL1. **A-C** Western blotting analyzes G3BP1 expression level after CHX treatment at 0, 2, 4, 6, 8 hours. **B-D** To quantify the G3BP1 protein signal in the lanes, line chart depicted the changes in G3BP1 in comparation with the two groups, KO-METTL1-T24/ctrl-T24 and OE-METTL1-SV-HUC-1/Plex-SV-HUC-1, in accordance with figure A and C, respectively.


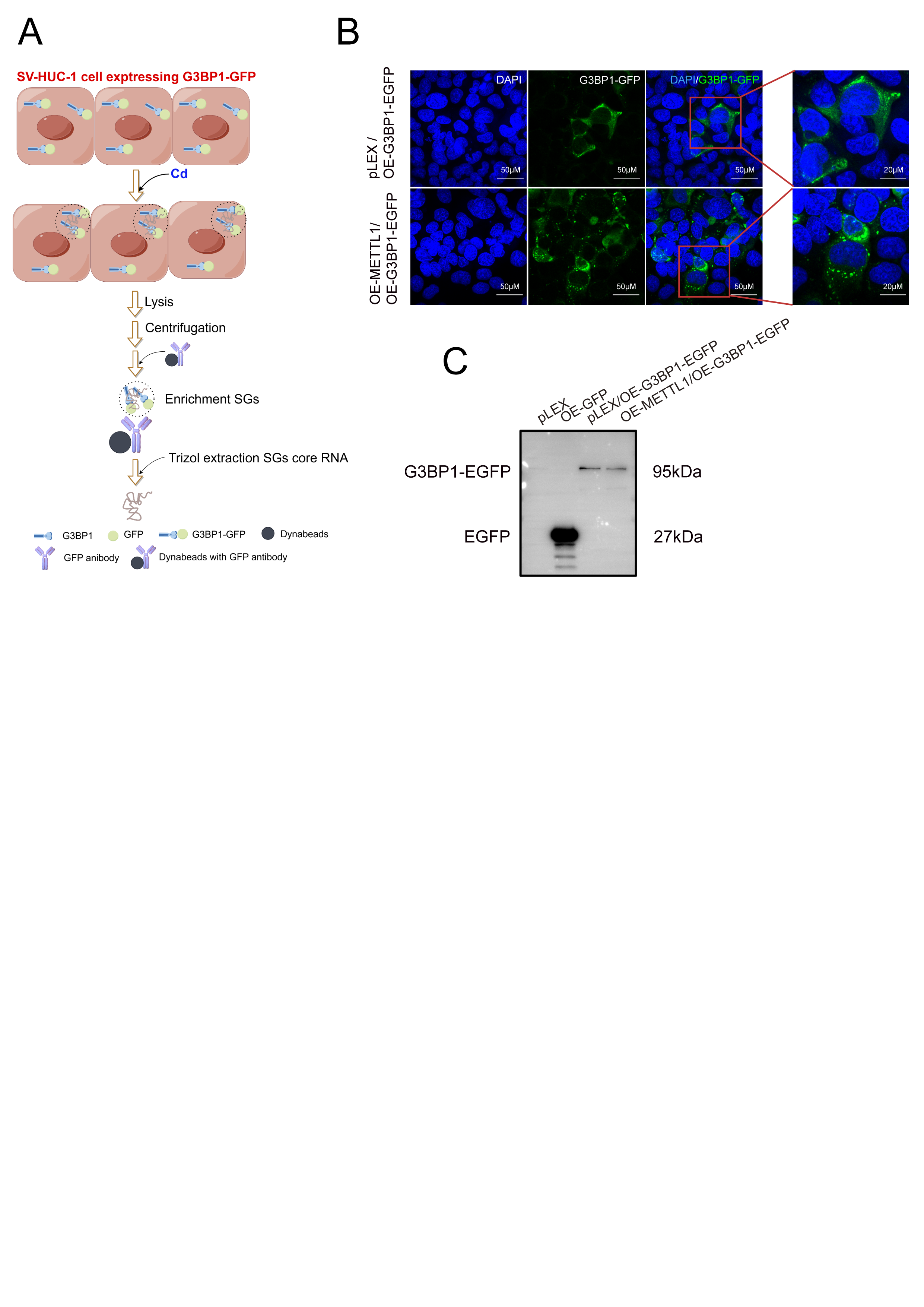


**Fig. S4** Extraction for SG core RNA. **A** The main steps for extracting the SG core RNA (Graphing website: https://www.figdraw.com/#/). **B** Fluorescence microscopy images of overexpressed-METTL1 SV-HUC-1 and control SV-HUC-1 cells expressing G3BP1-EGFP after exposure to CdCl_2_ (300μM, 1h). **C** Western blot verified the expression of G3BP1-EGFP in w/ and w/o overexpressed METTL1 SV-HUC-1 cells.


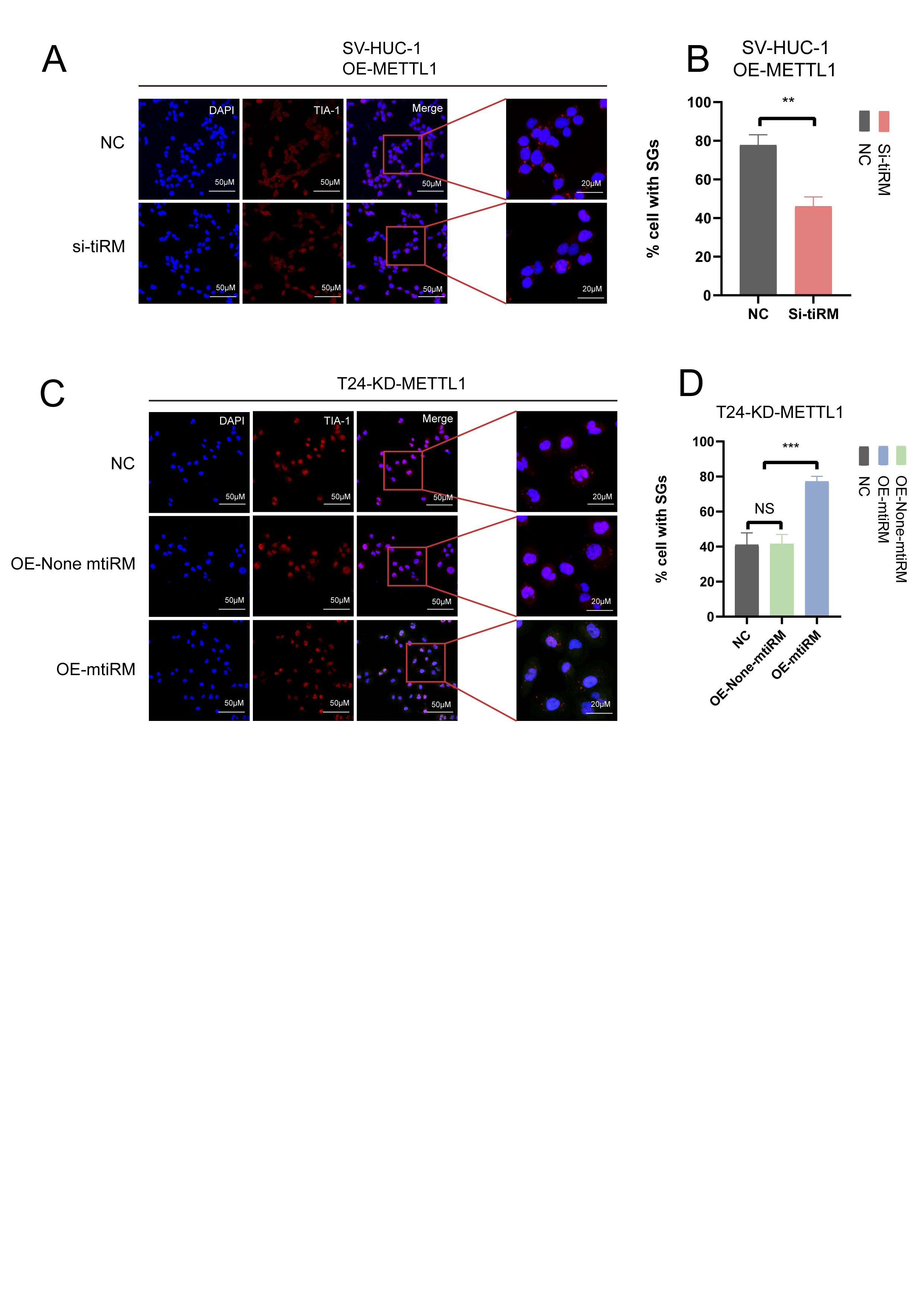


**Fig. S5** mtiRM regulates the formation of SGs. **A, B** METTL1-overexpressed SV-HUC-1 cells transfected tiRM inhibitor and NC were exposed to CdCl_2_ (300μM, 1h), and the percentage of positive cells for SGs was quantified (B). **C, D** T24-KD-METTL1 cells transfected tiRM mimics, endogenous mtiRM and NC were exposed to CdCl_2_ (300μM, 1h), and the percentage of positive cells for SGs was quantified (D). The data are representative of three independent experiments.

**Table S1.** The antibodies used in this study

| **Name** | **Manufacturer** |
| --- | --- |
| anti-METTL1 antibody | Abcam, ab220499 |
| anti-G3BP1 antibody | BD bioscience, 611126 |
| anti-β-actin antibody | Proteintech, 81115-1-RR |
| anti-GAPDH antibody | Proteintech, 10494-1-AP |
| Alexa Fluor 488 IgG antibody | Thermo, A21206 |
| Alexa Fluor 594 IgG antibody | Thermo, A11037 |
| anti-Flag antibody | CST, 2368S |

**Table S2.** The sequences of stem-loop Reverse Transciption (RT) primer and qPCR primer

| **Name** | **Species** | **sequence** |
| --- | --- | --- |
| METTL1 | Homo sapiens | Forward: GGCAACGTGCTCACTCCAA |
|  |  | Reverse:CACAGCCTATGTCTGCAAACT |
| β-Actin | Homo sapiens | Forward: CCTTGCACATGCCGGAG |
|  |  | Reverse: GCACAGAGCCTCGCCTT |
| G3BP1-EGFP | Homo sapiens | Forward: CCGACTCTACTAGAGGCCACCATGGTGATGGAGAA |
|  |  | Reverse: CCTCTAGACTCGAGCTTACTTGTACAGCTCGTCC |
| 3’tiRNA Met^CAT^ Forward Prime | Homo sapiens | ACACTCCAGCTGGGACAGTCCGACGATCTAATCTGAA |
| Universal Reverse Prime | Homo sapiens | CTCAACTGGTGTCGTGGAGTCGGCAATTCAGTTGAGTGGTTCCC |

**Table S3.** The sequences of probes used in this study

| **Name** | **sequence** |
| --- | --- |
| Inhibitor-3'tiRNA-Me^CAT^ | GGTCGTGAGTTCGATCCTCA |
| Mimic-3'tiRNA- Me^CAT^ | CAUAAUCUGAAGGUCGUGAGUUCGAUCCUCACACGGGGCACCA |
| Dig-3'tiRNA- Me^CAT^ | TGAGGATCGAACTCACGACC |
| Dig-U6 | TGGAACGCTTCACGAATTTG |

**Supplementary methods**

Plasmid construction and mutagenesis assay

Enzyme Digestion: The 2AB-Flag vector (4 µg) was digested using a reaction mixture containing 3 µl HindIII-HF, 3 µl EcoRI-HF, 5 µl CutSmart Buffer, and ddH₂O to a final volume of 50 µl. The mixture was incubated at 37°C for 3 hours to ensure complete digestion.

PCR Amplification: The G3BP1-Mut and G3BP1-WT fragments were amplified from the synthesized puc-GW-G3BP1-Mut and plvx-G3BP1-GFP plasmids, respectively, using KOD-PLUS-NEO Mix. The PCR program was as follows: initial denaturation at 94°C for 2 minutes, followed by 35 cycles of 94°C for 15 seconds, 50°C for 30 seconds, and 68°C for 45 seconds, with a final extension at 68°C for 7 minutes.

Gel Purification: The digested vector and amplified target fragments were separated by agarose gel electrophoresis to obtain fragments of the desired sizes. The relevant bands were excised from the gel, and the DNA was purified using a gel extraction kit according to the manufacturer's instructions.

Ligation: The purified digested vector and target fragments were ligated using the 2X Uniclone Seamless Cloning Mix (Uniclone Seamless Cloning Kit) under the following conditions: 50°C for 1 hour. The ligation mixture was then used to transform DH5α competent cells.

Transformation and Screening: The transformed DH5α cells were spread evenly on LB agar plates containing 100 µg/ml ampicillin and incubated at 37°C for 12 hours. Single colonies were picked and subjected to sequencing to verify the correct insertion of the G3BP1 fragments. Colonies with confirmed correct sequences were selected for further expansion.

Plasmid Extraction: Plasmids were extracted from the selected colonies using an endotoxin-free plasmid extraction kit, following the manufacturer's instructions. The extracted plasmids were stored at -20°C for subsequent experiments.

The buffers used in extraction for SG core RNA

①SG lysis buffer

| Components | Storage concentration | Volume |
| --- | --- | --- |
| 50 mM TrisHCl pH7.4 | 0.5M | 1mL |
| 100 mM KOAc | 1M | 1mL |
| 2 mM MgOAc | 0.5M | 40μL |
| 0.5 mM DTT | 1M | 5μL |
| 50 μg/mL Heparin | 0.05g/mL | 10μL |
| 0.5%NP40 | 10% | 500μL |
| Protease inhibitor | 200× | 50μL |
| 1U/μL RNasein Plus RNase Inhibitor | 40u/μL | 250μL |
| DEPC H_2_O |  | To 10mL |

②Wash buffer 1

| Components | Storage concentration | Volume |
| --- | --- | --- |
| 20 mM Tris HCl pH 8.0 | 0.5M | 400μL |
| 200 mM NaCl | 1M | 2mL |
| 1U/μL of RNasein Plus RNase Inhibitor | 40u/μL | 250μL |
| DEPC H_2_O |  | To 10mL |

③Wash buffer 2

| Components | Storage concentration | Volume |
| --- | --- | --- |
| 20 mM Tris HCl pH 8.0 | 0.5M | 400μL |
| 500 mM NaCl | 1M | 5mL |
| 1 U/μL of RNasein Plus RNase Inhibitor | 40u/μL | 250μL |
| DEPC H_2_O |  | To 10mL |

④ Wash buffer 3

SG lysis buffer with 2 M Urea

⑤ 1×Proteinase K buffer

| Components | Storage concentration | Volume |
| --- | --- | --- |
| 100 μg/mL Proteinase K | 20mg/mL | 1.25μL |
| 2 M Urea | 5M | 100μL |
| 1×TE buffer | 1× | 150μL |
| Total |  | 250μL |

The buffers used in Northern blot

| Buffer A | Stock | Vol for 100mL |
| --- | --- | --- |
| 5×SSC | 20× | 25mL |
| 0.1%Lauroyl sarcosine | 10%(wt/vol) | 1mL |
| 0.02%SDS | 10%(wt/vol) | 200μL |
| 1%BSA | 10%(wt/vol) | 10mL |
| DEPC H_2_O |  | To 100mL |

| High Stringent Buffer | Stock | Vol for 100mL |
| --- | --- | --- |
| 0.1×SSC | 20× | 500μL |
| 0.1%SDS | 10%(wt/vol) | 1mL |
| DEPC H_2_O |  | To 100mL |

| Low Stringent Buffer | Stock | Vol for 100mL |
| --- | --- | --- |
| 2×SSC | 20× | 10mL |
| 0.1%SDS | 10%(wt/vol) | 1mL |
| DEPC H_2_O |  | To 100mL |

| Washing Buffer | Stock | Vol for 100mL |
| --- | --- | --- |
| 1×SSC | 20× | 5mL |
| DEPC H_2_O | To 100mL | |

| Maleic acid Buffer | Stock | Vol for 250mL |
| --- | --- | --- |
| 0.1M maleic acid |  | 2.9g |
| 0.15M NaCl |  | 2.2g |
| DEPC H_2_O | pH7.5 | To 250mL |

| DIG washing Buffer | Stock | Vol for 500mL |
| --- | --- | --- |
| 0.1M maleic acid |  | 5.8g |
| 0.15M NaCl |  | 4.4g |
| 0.3%Tween 20 |  | 1.5mL |
| DEPC H_2_O | pH7.5 | To 500mL |

| Blocking reagent | Stock | Vol for 50mL |
| --- | --- | --- |
| BSA |  | 5g (10%) |
| Maleic acid Buffer | 4℃ | To 50mL |

| Development Buffer | Stock | Vol for 250mL |
| --- | --- | --- |
| 0.1M Tris-HCl |  | 3.025g |
| 0.1M NaCl |  | 1.45g |
| DEPC H_2_O | pH9.5 | To 250mL |

| Blocking Buffer | Stock | Vol for 10mL |
| --- | --- | --- |
| Blocking Reagent | 10% | 1mL |
| Maleic acid Buffer |  | To 10mL |
| 2×Stop Solution | Stock | Vol for 30mL |
| Deionized formamide |  | 28.5mL |
| 0.5M EDTA (pH8.0) |  | 1.2mL |
| Bromophenol blue |  | 15mg |
| Xylene cyanol |  | 15mg |
| DEPC H_2_O | pH9.5 | To 30mL |

| 2×Stop Solution | Stock | Vol for 30mL |
| --- | --- | --- |
| Deionized formamide |  | 28.5mL |
| 0.5M EDTA(pH8.0) |  | 1.2mL |
| Bromophenol blue |  | 15mg |
| Xylene cyanol |  | 15mg |
| DEPC H_2_O | pH9.5 | To 30mL |

The extraction of endogenous tiRNAs

1. SV-HUC-1 cells were seeded into six 10cm culture dishes in advance and allowed to grow overnight until the confluence reached 90%.
2. The cells were washed twice with 5-10 ml of 1× HBSS buffer to reduce RNase contamination from the culture medium.
3. After the second wash, the supernatant was discarded, and 1 ml of 1× HBSS buffer was added to each dish.
4. The cells were collected into 1.5ml EP tubes using a cell scraper, centrifuged at 1000 g for 3 min at 4°C, and the supernatant was removed as much as possible to prevent RNase contamination from the culture medium.
5. The cells were resuspended by pipetting with 250 μL of ANG digestion buffer, centrifuged at 1500 g for 30 s at 4°C, and the supernatant was transferred to a new 1.5ml EP tube.
6. Add angiogenin (ANG) to a final concentration of 100 nM to the supernatant, gently pipette to mix, and incubate at room temperature for 30 minutes.
7. Add 750 µL of Trizol reagent to extract RNA.
8. Subject the extracted RNA to SDS-urea denaturing polyacrylamide gel electrophoresis.
9. Transfer the gel to a solution of 3× SYBR Gel Red (diluted from 10000× SYBR Gel Red dye with DEPC-treated water), incubate on a rocker for 1-1.5 hours.
10. Excise the tiRNA fragment under UV light, add 1× Elution buffer to submerge the gel, incubate at 65°C for 15 minutes, freeze at -80°C for 15 minutes, incubate at 65°C for 15 minutes again, and then add Trizol to extract RNA.
11. Take 500 pmol of biotinylated oligo probe and heat at 90°C for 5 minutes to denature.
12. Add the denatured probe to the RNA, followed by 950 µL of TBS buffer, and transfer to a rotator for hybridization at room temperature for 1 hour.
13. Add 80 µL of streptavidin magnetic beads that have been washed three times with TBS buffer, transfer to a rotator, and incubate at room temperature for 1 hour.
14. Wash the magnetic beads three times with TBS buffer. Add Trizol to extract RNA.
15. Digest the probe with DNase, incubate at 37°C for 3 hours. Add Trizol to extract RNA.
16. Add m^7^G antibody to the RNA at a ratio of RNA:m^7^G antibody = 1:10, along with 500 µL of 1× IPP buffer, and incubate on a rotator at 4°C for 2 hours.
17. During incubation, wash the immunomagnetic beads three times with 1× IPP buffer, 5 minutes each.
18. After the third wash, discard the supernatant, resuspend the beads in 200 µL of 1× IPP buffer, and add the beads after incubation is complete.
19. Incubate on a rotator at 4°C overnight.
20. Wash the immunomagnetic beads three times with 1× IPP buffer. After the third wash, discard the supernatant, add Trizol to purify the RNA, and the resulting product is the endogenous 3'tiRNA with m^7^G modification.

tiRNA interference and overexpression

(1) Seed the cells in a 24-well plate in advance and allow them to grow overnight until the confluence reaches 40% to 50%. Before transfection, discard the old culture medium and replace it with fresh medium containing 10% FBS but without antibiotics.

(2) Transfect the Inhibitors using Lipofectamine RNAiMAX transfection reagent. The formulation is as follows:

|  | Components | Volume |
| --- | --- | --- |
| A tube | Inhibitors（10μM） | 1.5μL |
|  | Opti-MEM | 75μL |
| B tube | Lipofectamine RNAiMAX | 3.75μL |
|  | Opti-MEM | 75μL |

After mixing the contents of tubes A and B, let the mixture stand at room temperature for 5 minutes. Then, evenly drop it into the cell culture medium and incubate in a 37°C incubator for 48 to 72 hours.

(3) Transfect Mimics and endogenous tiRNA using Lipofectamine 3000 transfection reagent. The formulation is as follows:

|  | Components | Volume |
| --- | --- | --- |
| A tube | RNA | 500ng |
|  | Opti-MEM | 25μL |
|  | P3000 | 1μL |
| B tube | Opti-MEM | 25μL |
|  | Lipo3000 | 1μL |

After mixing the contents of tubes A and B, let the mixture stand at room temperature for 15 minutes. Then, evenly drop it into the cell culture medium and incubate in a 37°C incubator for 48 to 72 hours.

tiRNAs qRT-PCR

(1) 3'-Terminal Deacetylation Modification

① Prepare the reaction mixture.

| Components | Volume |
| --- | --- |
| Input RNA | ≦5μg |
| Deacylation Reaction Buffer(5X) | 3μL |
| RNase Inhibitor | 1μL |
| Nuclease-free Water | To 15μL |
| Total | 15μL |

② After a brief centrifugation to mix, incubate in a PCR machine at 37°C for 40 minutes.

③ Add 19 μL of Deacetylation Stop Buffer, mix by centrifugation, and then let stand at room temperature for 5 minutes.

④ Add Trizol to purify RNA.

(2) Demethylation Modification

① Prepare the reaction mixture.

| Components | Volume |
| --- | --- |
| Demethylation Reaction Buffer(5x) | 10μL |
| Demethylase | 5μL |
| RNase Inhibitor | 1μL |
| Input RNA | ≦5μg |
| Nuclease-free water | To 50μL |
| Total | 50ul |

② After a brief centrifugation to mix, incubate in a heating block at 37°C for 2 hours.

③ Add 40 μL of nuclease-free water and 10 μL of Demethylation Stop Buffer (5X), mix by centrifugation, and then let stand at room temperature for 5 minutes.

④ Add Trizol to purify RNA.

(3) 3'-Terminal Dephosphorylation

① Prepare the reaction mixture.

| Components | Volume |
| --- | --- |
| Input RNA | ≦3μg |
| 10x T4 DNA ligase buffer | 5μL |
| T4 PNK | 3μL |
| RNase inhibitor | 1μL |
| Nuclease-free water | To 50μL |
| Total | 50μL |

② After a brief centrifugation to mix, incubate in a PCR machine at 37°C for 40 minutes, followed by 65°C for 20 minutes.

③ Add Trizol to purify RNA.

(4) 5'-Terminal Adaptor Ligation

① Prepare the reaction mixture.

| Components | Volume |
| --- | --- |
| 10× T4 RNA ligation buffer | 5μL |
| Input RNA | ≦3μg |
| ATP | 5μL |
| RNA ligase | 1μL |
| 5’ adaptor | 5μL |
| RNase inhibitor | 1μL |
| Nuclease-free water | To 50μL |
| Total | 50μL |

② After a brief centrifugation to mix, incubate in a PCR machine at 37°C for 45 minutes, followed by 65°C for 15 minutes.

③ Add Trizol to purify RNA.

(5) Reverse Transcription

① Prepare the reaction mixture according to the Takara RR037 reverse transcription kit.

| Components | Volume |
| --- | --- |
| 5x PrimeScipt Buffer | 4μL |
| Enzyme Mix I | 1μL |
| Special Prime | 2μL |
| Input RNA | 12μL |
| RNase inhibitor | 1μL |
| Total | 20μL |

Special Prime:

A. The reverse transcription primers used in this step are universal. The sequences are as follows:

| Name | Sequence（5’ to 3’） |
| --- | --- |
| 3’tiRNA MetCAT Forward Prime | ACACTCCAGCTGGGACAGTCCGACGATCTAATCTGAA |
| Universal Reverse Prime | CTCAACTGGTGTCGTGGAGTCGGCAATTCAGTTGAGTGGTTCCC |

B. Dilute the reverse transcription primers to a concentration of 10μM, and mix them in equal proportions to achieve a final concentration of 1μM.

② After a brief centrifugation to mix, place in a PCR machine with the program set at 16°C for 30 minutes, 42°C for 30 minutes, 85°C for 55 seconds, and then hold at 4°C indefinitely.

(6) Detection was performed using a qRT-PCR instrument.

Ribosome profiling sequencing (Ribo-seq)

T24 V2 and T24-KD-METTL1 cells were cultured to a density of 10^7 cells and treated with 100 μg/ml CHX (Sigma) for 15 minutes. Subsequently, Ribo-seq was performed using the TruSeq Ribo Profile kit (Illumina). The ribosome-protected fragments (RPFs) were generated through nuclease digestion and subsequently purified using the RNA Clean & Concentrator-25 kit (Zymo Research, Cat. #: R1017). Additionally, rRNA was depleted using the Ribo-Zero Gold kit (Illumina, Cat. #: MRZG12324). Both the purified RPFs and fragmented input RNA samples were then processed for library construction with the NEBNext Multiple Small RNA Library Prep Set for Illumina (New England Biolabs, Cat. #: E7300L) and sequenced on an Illumina HiSeq X Ten platform. The Ribo-seq data were analyzed as previously described.
