# Supplementary material for: METTL1 promotes cadmium-induced stress granules formation via enhancing translation of G3BP1 and expression of m7G- 3' tiRNA MetCAT
Source: Cell Biol Toxicol. 2025 Aug 5;41(1):124. doi: 10.1007/s10565-025-10072-0 (PMC12325392; doi:10.1007/s10565-025-10072-0)
Supplement: Supplementary file 7 — Supplementary file7 (PDF 137 KB) [file 10565_2025_10072_MOESM7_ESM.pdf]

AZENTA LIFE SCIENCES

Certificate of Analysis

Project Tracking #	Quote #	Clone ID #
80-1123889315	80-1123889315_R0	LA23710-3/Y700856

Sequence Name	Cloning Vector
G3BP1 CDS m7G mut in pUC-GW-Amp	pUC-GW-Amp

Cloning Sites	Insert Length
NA	1401

QC Item	Specification	Results
Insert Sequence	Insert sequence results consistent with target	√ Pass
Vector Sequence	Flanking sequence consistent with expected	√ Pass
Open Reading Frame across junction	Correct and consistent with target	n/a
Restriction Digest	Expected fragment sizes observed	√ Pass
PCR amplification	Correct without non-specific bands	n/a
DNA Quantity and Quality	Actual yield (by A_{260})	4 ug
	Concentration (n/a if lyophilized)	n/a
	Purity ($A_{260/A280}$ = 1.8 - 2.0)	√ Pass
	# of tubes	1
	Matrix	TE (lyophilized)
Endotoxin Test	Verified, ＜0.1 EU/ug(endo-free preps only)	n/a
Appearance	Clear, no visible particles	√ Pass
Label	Correct and clear	√ Pass
Comments		

Restriction Digest Test Results

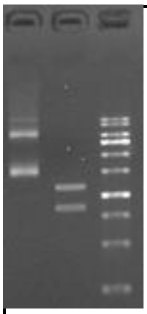

Lane Description(from left to right)
Lane 1: undigested
Lane 2: digested with PvuII
Lane 3: ladder

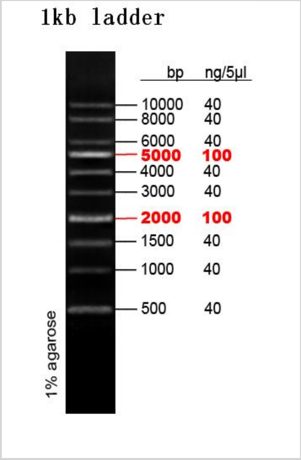

Certified By
Yan Li

Date
02/14/2023
